# Implicit Prosody and Cue-based Retrieval: L1 and L2 Agreement and Comprehension during Reading

**DOI:** 10.3389/fpsyg.2016.01922

**Published:** 2016-12-15

**Authors:** Elizabeth Pratt, Eva M. Fernández

**Affiliations:** ^1^The Graduate Center, City University of New YorkNew York, NY, USA; ^2^Queens College and Graduate Center, City University of New YorkNew York, NY, USA

**Keywords:** prosody, implicit prosody, agreement, comprehension, cue-based retrieval, L2 reading

## Abstract

This project focuses on structural and prosodic effects during reading, examining their influence on agreement processing and comprehension in native English (L1) and Spanish–English bilingual (L2) speakers. We consolidate research from several distinct areas of inquiry—cognitive processing, reading fluency, and L1/L2 processing—in order to support the integration of prosody with a cue-based retrieval mechanism for subject-verb agreement. To explore this proposal, the experimental design manipulated text presentation to influence implicit prosody, using sentences designed to induce subject-verb agreement attraction errors. Materials included simple and complex relative clauses with head nouns and verbs that were either matched or mismatched for number. Participants read items in one of three presentation formats (whole sentence, word-by-word, or phrase-by-phrase), rated each item for grammaticality, and responded to a comprehension probe. Results indicated that while overall, message comprehension was prioritized over subject-verb agreement computation, presentation format differentially affected both measures in the L1 and L2 groups. For the L1 participants, facilitating the projection of phrasal prosody onto text (phrase-by-phrase presentation) enhanced performance in agreement processing, while disrupting prosodic projection via word-by-word presentation decreased comprehension accuracy. For the L2 participants, however, phrase-by-phrase presentation was not significantly beneficial for agreement processing, and additionally resulted in lower comprehension accuracy. These differences point to a significant role of prosodic phrasing during agreement processing in both L1 and L2 speakers, additionally suggesting that it may contribute to a cue-based retrieval agreement model, either acting as a cue directly, or otherwise scaffolding the retrieval process. The discussion and results presented provide support both for a cue-based retrieval mechanism in agreement, and the function of prosody within such a mechanism, adding further insight into the interaction of retrieval processes, cognitive task load, and the role of implicit prosody.

## Introduction

The computation of agreement has been examined extensively in both formal theoretical and experimental settings, and studies have identified many features that contribute to the processing of agreement in both production and comprehension. Despite the relatively uninformative nature of subject-verb agreement in English, it is nonetheless calculated almost automatically, with agreement errors triggering processing difficulty and rapid reanalysis effects (Pearlmutter et al., 1999). Also, even in the case of English, where the agreement system is comparably simple, errors still occur during production (Bock and Miller, 1991), and may go unnoticed during comprehension (Nicol et al., 1997). The circumstances under which these effects may occur provide compelling insight into the computation of agreement, and how it may proceed online. While agreement is often considered a primarily syntactic computation, it also integrates information from non-syntactic sources, which makes it an ideal testing ground to investigate syntactic processing and general processing strategies.

Agreement phenomena have been studied in L2 speakers as well, albeit not as extensively. There is conflicting evidence on how L2 speakers process agreement, with some reports of native-like sensitivity to errors (Tanner, 2011) and others of relative insensitivity to errors (Jiang, 2004; Keating, 2009). Models of overall L2 processing also tend to diverge in their predictions of performance and sources of non-convergence with L1 speakers—a central question being whether L2 processing differences are primarily representational or performance-based.

The online use of prosody in agreement processing is also relatively underexamined. This is particularly the case for agreement processing during silent reading, where there are no direct methods of measuring how prosody is being used, or whether it functions similarly as in oral production and comprehension. The effect of segmented text on comprehension has been explored within the pedagogical and reading subfields, but has not been explicitly linked with the observed effects of implicit prosody, or extensively adapted for psycholinguistic research. The experiments presented here take a step in this direction, applying text segmentation as a way of tapping into the role of (implicit) prosody during reading and exploring how it interacts with comprehension and grammatical processing—specifically, agreement in complex relative clause constructions. We use this evidence to advance the proposal that prosodic phrasing may act as a cue within a cue-based memory retrieval system for agreement.

## Background

### Agreement and Attraction

Over the last decades, subject-verb agreement has been widely studied as a window into the mechanisms of real-time sentence processing. Despite the morphological poverty of the agreement system in English, speakers nonetheless produce errors in both oral and written production (Bock and Miller, 1991; Bock and Cutting, 1992), with similar rates of error detection in comprehension (Pearlmutter et al., 1999; Wagers, 2008). Regardless of its informativeness, agreement is consistently computed in English, with violations of agreement resulting in processing difficulty (Pearlmutter et al., 1999), and triggering neural responses to subject-verb mismatches (Kaan, 2002). The study of agreement computation in general is thus an effective measure of online sentence processing, which allows for further inference into the general mechanisms of production and comprehension.

Much of the current research into the agreement mechanism has drawn from Bock and Miller’s (1991) seminal work on agreement attraction. In an elicited production task, Bock and Miller presented participants with an auditory preamble consisting of a subject phrase as in (1). Participants were instructed to repeat back the preamble and complete the sentence.

(1) (a) The key to the cabinets…
     (b) The keys to the cabinet…

The critical finding of this study was that errors were far more likely to occur when the local noun was plural (e.g., 1a) than when it was singular (e.g., 1b). Following these results, as well as those of Bock and Cutting (1992), Bock and colleagues proposed the following characteristics of subject-verb agreement errors: (i) verb number errors are more likely to occur when the head noun and local noun are mismatched for number—almost exclusively when a singular head noun is followed by a plural local noun (*plural markedness effect*); (ii) errors are more likely in complex subject constructions with a PP modifier than with a relative clause modifier (*clause boundary effect*); and (iii) conceptual number and animacy have little effect on the number of errors produced.

This initial set of studies set the stage for subsequent investigations into the types of information that contribute to agreement processing. Subsequent studies have found effects of both linear and hierarchical distance (Nicol, 1995; Vigliocco and Nicol, 1998), distributivity and notional number (Vigliocco et al., 1996a,b), and plausibility factors (Thornton and MacDonald, 2003), not only in production, but in comprehension as well (Freedman and Forster, 1985; Osterhout and Mobley, 1995; Nicol et al., 1997; Clifton et al., 1999; Pearlmutter et al., 1999; Häussler and Bader, 2009). While the mechanism of agreement is often viewed as primarily syntactic, these studies demonstrate that agreement computation integrates information from multiple sources. Semantic, phonological, and pragmatic information contribute to how agreement and number features are calculated, in addition to syntactic and morphological information. Since agreement phenomena are not confined to syntactic factors, it is argued that there must be processing constraints which allow efficient computation in real time, and which can shed light on how and why agreement errors occur.

### L2 Agreement and Attraction

Research into L2 morphological processing has also provided rich data on L2 processing profiles. Generally speaking, adult L2 learners are not always sensitive to morphological features during comprehension—e.g., tense, number, and agreement (Johnson et al., 1996; Lardiere, 1998). Difficulties seem to persist despite advanced proficiency, high L2 exposure, and equivalent features in the L1 (Hopp, 2007). Much L2 morphological processing research examines L2 speakers’ sensitivity to morphological features during comprehension, and whether divergence from native speakers is due to representation (competence) or processing (performance). There is much support for a processing-based approach, where proficiency (Foote, 2011) and processing load (Keating, 2009) appear to be better predictors of native-like patterns. Work by Kilborn (1992) also suggests that task demand and the resulting limitation in resources is a critical factor in L2 sentence processing.

In a word-monitoring task, Kilborn measured the online integration of syntactic and semantic information by L1 and L2 English speakers in three conditions: (i) a ‘complete’ sentence condition, where both syntactic and semantic information were available, (ii) a ‘syntactic’ condition where syntactic structure was maintained but nouns and verbs were replaced and semantic cues were missing, and (iii) a ‘random’ word string condition where both syntactic and semantic cues were missing. The L2 group performed the task under normal conditions in both their L1 (German) and their L2 (English), while the native English group performed the task in normal and noise conditions.

When performing the task in their L1, the L2 group was faster at detecting the target word in the syntactic condition than in the random condition, and fastest in the complete condition. However, when performing the task in the L2, they were facilitated by the presence of syntactic information (‘syntactic’ condition), but were not further benefited by semantic information in the complete condition. The native speakers mirrored these patterns in their normal and noise test conditions. In the normal condition, they responded to the target word most quickly in the complete condition, then the syntactic, then the random condition. In the noise condition, native speakers were facilitated by syntactic structure, but were not further facilitated by the addition of semantic information in the complete condition. These results indicate that task demand affects processing speed and automaticity, and interestingly, that even native speakers can look like learners when under sufficient processing burden.

McDonald (2006) further strengthened this argument for general processing-based differences by comparing native and L2 speakers’ performance on several processing measures, including a grammaticality task. While L2 speakers performed significantly worse than native speakers in detecting agreement errors, a second experiment demonstrated that they performed comparably to native speakers completing the task under various noise conditions such as digit load, white noise, timed response, or compressed speech.

Tanner (2011) investigated agreement attraction effects in native speakers and L2 learners of English, measuring behavioral and ERP responses to constructions containing grammatical or ungrammatical PP subject modifiers (2) and relative clause subject modifiers (3).

(2) The winner of the big trophy/trophies has/have proud parents.
(3) The winner who received the trophy/trophies has/have proud parents.

Behavioral results and ERP response profiles indicated that the L2 learners’ responses patterned with those of native speakers. The difference between native speakers and L2 learners was found to be largely quantitative rather than qualitative, and Tanner found correlations particularly with L2 proficiency, age of arrival, and motivational factors.

Other studies have supported these findings, suggesting that while L2 speakers may appear to be less sensitive to errors than L1 speakers (Jiang, 2004, 2007; Sato and Felser, 2006; Keating, 2009), this difference is mediated by L2 proficiency rather than qualitative processing differences (Hopp, 2007; Foote, 2011).

Given the evident similarities between L1 and L2 performance, factors seemingly unique to second language acquisition such as proficiency and online processing capacity could be reframed as general processing load effects, thereby treating L2 differences as quantitative effects rather than qualitative ones.

### Theoretical Accounts of Agreement

Returning to the computation of agreement, a number of theoretical accounts have been proposed to accommodate the findings summarized earlier, including feature percolation, marking and morphing, feature selection/feature copy, and semantic integration, which we summarize briefly below.

In the feature percolation account (Nicol et al., 1997; Vigliocco and Nicol, 1998; Franck et al., 2002), features of a noun phrase can percolate upward through the syntactic structure and erroneously value a noun higher in the structure. The greater the structural or hierarchical distance between the attractor and head noun, the lower the likelihood of feature percolation. The model is able to account for both clause boundary effects and plural markedness effects: the greater the structural distance between the two nouns, the lower the likelihood of feature percolation; and the more marked a feature (e.g., *plural*), the greater the likelihood of it overwriting a less marked feature (e.g., *singular*). However, the model predicts that percolation effects should occur regardless of the grammaticality of the sentence: some ungrammatical sentences may appear grammatical, and some grammatical sentences may appear ungrammatical. Contrary to this prediction, results obtained by Wagers et al. (2009) indicate that while ungrammatical constructions can appear grammatical, ‘illusions of ungrammaticality’ in sentences that are actually grammatical are exceedingly rare. This strongly suggests that agreement attraction is not due to the erroneous representation of the subject via percolation (Wagers, 2008; Wagers et al., 2009).

The Marking and Morphing model (Eberhard et al., 2005), was originally proposed as an elaboration of the percolation model, and describes the agreement mechanism as two primary procedures, *marking* and *morphing*. Marking, which maps conceptual number information onto syntactic elements (e.g., the subject NP), and morphing, which is the structural integration of the elements, during which the verb inherits number from the subject. Based on evidence that conceptual/notional features of the head noun influence agreement while those of the attractor noun do not (Eberhard, 1999; Bock and Middleton, 2011, among others), attraction is said to occur during morphing, when number features are integrated within the noun phrase, and the resulting feature is conveyed to the verb. This model has been brought under scrutiny, however, by Franck and colleagues (see Franck, 2011) for its inability to account for finer aspects of the syntactic structure (such as c-command and precedence distinctions) that modulate the strength of agreement attraction, as well as for inadequately explaining attraction across clause boundaries.

In partial response to the Marking and Morphing model, Franck et al. (2008) proposed integrating theoretical syntax with lexical production processes, suggesting that agreement is composed of two primary operations—*feature selection*, which operates within the lexicon, selecting number features according to conceptual input, and even morphophonological features, and *feature copy*, which operates within the syntax as an AGREE function that transmits number features to the verb. Under this model, agreement errors are found to be sensitive to c-command/precedence relations, both during and after the structural derivation process. As a sentence is built, certain configurations—such as precedence of the attractor or lack of Spec-head agreement—may increase the likelihood of a verb copying the features of an attractor rather than the subject. However, this model, while acknowledging semantic and conceptual input during feature selection, does not incorporate these non-syntactic sources into the error model, which occurs wholly within the syntax alone.

Solomon and Pearlmutter (2004) focus on the conceptual and semantic relationship between nouns, which they refer to as “semantic integration.” For agreement processing in production, they propose that constructions containing more related/integrated elements are more susceptible to error, since the subject noun and local noun are activated in the parser at the same time, and thus may interfere with each other during grammatical mapping. In the case of a number mismatch between the head and local noun, the incorrect verb form may be chosen. While the semantic integration account is able to handle several critical features of agreement attraction, it cannot accommodate the robust plural markedness effect, nor the difference in attraction effects in PP and relative clause constructions.

In relation to agreement attraction in comprehension, Pearlmutter et al. (1999) proposed that number mismatches between the nouns increase interference, and thus increase the likelihood of erroneous feature matching/retrieval of the subject NP at the verb. They suggest that an alternative model would allow number features to attain a level of activation during processing, allowing checking of those features when the verb is reached. However, the interference of intervening elements (particularly competing elements with number features of their own), would increase the rate of NP-mismatch effects, as well as grammaticality effects, as the number of the verb does not match that of the retrieved head number feature.

While agreement has been approached from these and other standpoints, other recent accounts contend that the architecture for its computation is similar to that of general memory and retrieval—specifically a cue-based content-addressable system (Van Dyke and Lewis, 2003; Lewis and Vasishth, 2005; Wagers, 2008; Wagers et al., 2009). Following key points in Solomon and Pearlmutter (2004), Wagers et al. (2009) argue against representation-based theories of attraction such as feature percolation, noting that this model in particular generates several testable predictions that are critical to its viability: (i) strong attraction effects should not occur in constructions where the local noun is not directly dominated by the head, and where it does not intervene between that head and the agreeing verb; (ii) attraction effects should occur regardless of whether the construction is grammatical or not. Wagers et al. (2009) examined these predictions via a series of self-paced reading and RSVP experiments, arriving at several critical conclusions: (i) both ungrammatical materials and plural elements are read more slowly, indicating that they incur a greater processing cost; (ii) ungrammatical materials are read more slowly than grammatical materials, even when the attractor does not intervene hierarchically between the subject and the verb; (iii) attraction effects are observed in ungrammatical, but not grammatical sentences (i.e., ungrammatical sentences may appear grammatical, but not vice versa).

Based on these results, Wagers et al. (2009) argue for an agreement system that is not based on erroneous number representation of the subject (either by percolation or other means), but on a cue-based retrieval mechanism triggered at the verb itself. This type of mechanism—discussed and developed by Lewis and Vasishth (2005) and McElree (2006)—is triggered at the verb after detection of a subject-verb mismatch, and initiates retrieval of the subject in memory.

Cue-based memory retrieval works within syntactic phrase structure configurations but does not order its feature-match process by hierarchical relations. Instead, it uses a probe-goal retrieval to specifically isolate constituents with a particular set of features. This allows for the rapid resolution of dependencies needed for online processing (McElree et al., 2003), but also accommodates error patterns found in production and comprehension where, based on feature overlap, multiple candidate constituents may be retrieved, even if not all are grammatically licensed.

### Cue-based Memory Retrieval

The cue-based retrieval model adopted by Wagers (2008) and Wagers et al. (2009) is based on proposals developed for both production (Badecker and Kuminiak, 2007; Badecker and Lewis, 2007) and comprehension (McElree et al., 2003; Lewis and Vasishth, 2005). Across the distinct instantiations of the models developed to date, they are consistent in emphasizing the critical role of memory within linguistic processing; specifically, that elements must be maintained in memory and retrieved at a relevant point for integration and interpretation (Chomsky and Miller, 1963; Miller and Chomsky, 1963; Wanner and Maratsos, 1978; MacWhinney, 1986; Abney and Johnson, 1991, among many others). Thus, language is not treated as an isolated processing module, but rather is affected by general constraints on cognition rooted in the memory system, such as activation levels, decay, and rehearsal (Lewis and Vasishth, 2005).

In relation to language processing, there is a two-part structure of attentional focus and memory store. The attentional focus can contain a single chunk at a time (McElree, 2001; McElree et al., 2003); as items come in, previous items are moved from attentional focus into the memory store. When an incoming item must reference or integrate with items outside of the current focus, those items must be retrieved from the memory store. This retrieval takes place via a cue-based system, whereby the specific features or cues of an item are targeted for retrieval.

Following the discussion in Wagers (2008), the retrieval system for agreement may function in two directions: both bottom-up and top-down, and can predict what features an agreeing element should have. For example, a subject marked with particular features may prime the system to expect a verb with matching features: a singular subject may be marked with a set of morphosyntactic features such as number, Case (and others such as gender, if relevant), and perhaps semantic cues such as Animate. While processing, the comprehender reaches the subject, which is marked with features that allow prediction of the upcoming verb form. If the incoming verb matches the retrieved prediction, it is integrated with the previous material, and processing continues. In the case of a mismatch, a retrieval mechanism initiates a search in the memory store, resulting in increased processing time compared to a match. If the retrieval operation finds a partial match in the memory store, the ungrammatical detection effect may be mitigated or even eliminated. Once items are processed, they begin to rapidly decay in storage, making retrieval more difficult as more time passes between presentation of subject and verb. However, rehearsal improves retention and elements may be reactivated at particular critical nodes, strengthening their signal before decay and making attraction effects less likely in certain structures than others (Lewis and Vasishth, 2005; Badecker and Kuminiak, 2007).

This interaction of decay and reactivation, along with the initial feature match strength, forms the basis of the retrieval model for agreement. Both syntactic and semantic/pragmatic cues can influence retrieval (Van Dyke and McElree, 2011), which allows not only for similarity-based interference on a lexical-featural level, but also frequency effects of lexical items or structural forms, which may account for processing asymmetries not accounted for by activation and decay alone. Wagers (2008) and Wagers et al. (2009) suggest that structural features may also be incorporated as feature-cues—a proposal further explored in Franck and Wagers (2015). Combining the effects of hierarchy with structural position and semantic cues thus provides a robust system for encoding structure into the retrieval process.

In relation to agreement, we assume that all speakers use a similar retrieval mechanism, regardless of L1/L2 language profile. While many prominent models of L2 processing assume either a different architecture from L1 or an inability to attain native-like performance, we assume that cue-based retrieval is a general cognitive strategy available to speakers of all languages and proficiency levels. In support of this recent work by Johns et al. (2015) demonstrates that direct retrieval is not exclusive to skilled readers, but is available to low-proficiency readers as well. Further, evidence from Lago et al. (2015) indicates that not only do attraction effects occur in similar distribution patterns in both Spanish and English, but also that a cue-based retrieval mechanism is plausibly deployed in both languages.

Variations of the cue-based retrieval mechanism have been successfully applied to agreement and memory during processing (see Wagers et al., 2009; Tanner, 2011)^1^. Since prosodic phrasing and implicit prosody—the projection of phrasing rhythm onto text during silent reading—have been found to affect processing strategies and parsing preferences, it is likely that prosodic cues can be incorporated into the model as well. Relating to agreement, the role of prosody may enhance memory either by interacting directly with the memory system, or indirectly via memory for structure. The prosodic contour may give additional cues to the memory system, or reinforce particular cues as relevant to the parse of the structure. Disruption of the prosodic projection during processing may further impair detection of ungrammaticality in complex sentences.

### Prosody and Processing

Prosodic boundaries, as indicated by pauses between phrases, play a considerable role in interpretation, and prosody in general has been shown to influence multiple aspects of auditory processing and comprehension: beginning with segmentation of the speech stream, and continuing further to the disambiguation of syntactic structures, and facilitation of memory. The Implicit Prosody Hypothesis (Fodor, 1998, 2002) proposes that readers project a “default” prosody onto text which, among other functions, allows them to better track dependency relations, particularly across long distances.

Building on this view, Kreiner (2005) proposed that natural reading prosody facilitates online syntactic integration, allowing subject-verb mismatches to be more easily detected. However, if this integration is disrupted in some way, mismatches will be more difficult to detect. Testing this hypothesis, Kreiner (2005) found no significant effect when the subject and verb were adjacent. However, when the subject and verb were separated by a relative clause, agreement errors were detected when the participants read with natural prosody, but not when prosody was disrupted. These results suggest that natural prosody does facilitate subject-verb agreement processing, particularly when processing and/or working memory load is greater.

Directly relating to the current study, Cromer (1970) found that segmentation of text variably influences readers’ comprehension based on reading proficiency. While the skilled readers in his study were generally resistant to manipulation of text presentation format, both intermediate and poor readers were affected. Intermediate readers performed best when text was presented in phrasal segments, but their comprehension was disrupted when text was presented word-by-word, or in non-phrasal fragments. On the other hand, low-proficiency readers performed best when text was presented word-by-word, but were not affected by any other presentation format.

Other researchers have also noted the correlation of fluency with comprehension, noting that greater reading fluency is associated with more ‘appropriate’ prosodic phrasing and contours (Clay and Imlach, 1971; Dowhower, 1987; Rasinski et al., 1994; LeVasseur et al., 2006; LeVasseur et al., 2008). Prosody may be seen as an intermediary between fluency and comprehension, such that individuals who demonstrate appropriate prosody are more likely to exhibit better comprehension as well (Paige et al., 2014).

### L2 Prosody and Text Segmentation in Reading

Looking at major prosodic features crosslinguistically, languages perhaps most notably differ in intonation and lexical stress patterns (see discussion in Cutler, 2012). However, general prosodic phrasing patterns appear to be rather universal, at least when those patterns align with syntactic constituents. There is also evidence that L2 learners are able to use both auditory and written prosodic cues to disambiguate structures, and revise initial parses. Dekydtspotter et al. (2008) examined L2 relative clause attachment ambiguities using both aural and written stimuli with both beginner and intermediate L2 French learners. Their results demonstrated that not only can learners make use of prosodic cues, but that they are also capable of deploying and integrating syntactic and prosodic strategies during parsing. In some cases, L2 speakers may rely more heavily on prosodic rather than syntactic cues, particularly when syntax and prosody are misaligned (Harley et al., 1995).

Relatedly, in a series of studies with Japanese learners of English, Kadota (1982) and Kadota and Tada (1992) found that text segmented into phrasal units improved comprehension and recall rates over sentence or word unit presentations (see Yamashita and Ichikawa, 2010). In further work, Yamashita and Ichikawa (2010) found that presentation formats which deliberately interrupted grammatical phrases or units disrupted comprehension for lower proficiency learners, but not for the more advanced learners. The results suggest that the advanced learners’ typical phrasing patterns may override the effect of text presentation, but lower proficiency learners’ underdeveloped phrasing patterns make their reading more susceptible to disruptive (or facilitative) effects. Evidence would suggest, then, that where the performance of L1 and L2 speakers may diverge is not necessarily in prosodic phrasing itself, but in its relation to overall fluency effects and the availability of processing resources.

There is, of course, concern as to whether phrasing effects during reading directly correspond to similar prosodic effects in auditory comprehension. Some previous studies investigating presentation formats have attributed processing differences to either purely syntactic factors, e.g., whether phrasal breaks are given at syntactic boundaries or not, or to the disruptive effect of word-by-word presentation, which prevents typical reading behaviors such as parafoveal preview and regressive eye movements to earlier material (Rayner et al., 2012). However, other studies have closely linked presentation format with prosodic phrasing during reading (Rasinski et al., 1994; LeVasseur et al., 2006, 2008). Further, the appropriate insertion of punctuation in both simple and complex sentences facilitates reading (Hirotani et al., 2006; Staub, 2007), suggesting that they act as cues to implicit prosodic boundaries. Taken together, it is not unreasonable to assume that manipulation of text format can directly disrupt the projection of a prosodic contour during silent reading.

The integration of prosody, particularly prosodic phrasing, into a cue-based retrieval model has not been addressed to date, although it is well motivated. In speech production, pauses tend to occur at clause boundaries, suggesting that clauses typically function as planning units (Butterworth, 1980; Garrett, 1982; McDaniel et al., 2010). While prosody is arguably not a primary information source during parsing, it clearly provides ancillary support that aids processing. When prosody is disrupted, greater strain is placed on memory and memory-based tasks, an effect we expect to observe most robustly in complex and demanding parsing contexts. Assuming that prosody plays a role in structural memory, it would likely interact with a cue-based memory retrieval mechanism as outlined above.

## Study Design

To build empirical support for the role of prosody within a cue-based retrieval model, we investigate the interactions of structural complexity and text presentation on comprehension and agreement processing during reading in L1 and L2 speakers of English. Grammatical and ungrammatical relative clause constructions of two complexity levels (Simple, Complex) were presented in one of three formats: whole sentence, word-by-word, and phrase-by-phrase. Both L1-English and L1-Spanish/L2-English speakers provided response times, grammaticality ratings, and sentence comprehension responses for probes targeting either general message comprehension or relative clause interpretation.

### Proposal and Overview of Predictions

For the current data, we predicted that agreement error detection would be lower for complex items versus simple items, and lower for the L2 participants versus the L1 participants, due to task load, memory load, or other factors.

The presentation format allows us to explore how text segmentation modulates implicit prosody, and whether implicit prosody plays a role during reading. Prosody could be beneficial to grammatical processing because it aligns with the syntactic structure and/or reduces memory load. When presentation format disrupts processing, it may be the result of conflict with the prosody projected onto the text by the reader.

A previously run pilot study included both subject- and object-relative clauses, manipulating both attractor number and verb number (sample set given in **Table 1**). Forty-six native English speakers were recruited from the Queens College psychology subject pool, and responded to grammaticality and comprehension prompts for each item (following the same experimental paradigm for the current study, described below). ANOVAs performed on grammaticality ratings indicated that overall, grammatical sentences were rated significantly higher than ungrammatical sentences [*F*(1,45) = 20.27, *p* < 0.001]. However there was a significant interaction between grammaticality and attractor number [*F*(1,45) = 5.25, *p* < 0.05], such that while there was no significant difference within the grammatical condition, in the ungrammatical condition, plural attractor items were rated significantly higher than singular attractor items (*p* < 0.01). This aligns with previous studies that have found a significant attraction effect only for ungrammatical plural attractor items (see, in particular, Wagers et al., 2009).

**Table 1:** Pilot materials – Subject relative clause sample set.

	Head	Attractor	Verb	Sentence
Grammatical	Singular (S)	Singular (S)	Singular (S)	(a) S–S–S: The reporter who called the senator writes…
	Singular (S)	Plural (P)	Singular (S)	(b) S–P–S: The reporter who called the senators writes…
Ungrammatical	Singular (S)	Singular (S)	Plural (P)	(c) S–S–P: ^∗^The reporter who called the senator write…
	Singular (S)	Plural (P)	Plural (P)	(d) S–P–P: ^∗^The reporter who called the senators write…

*^∗^indicates ungrammaticality.*

For the current study, all items were subject relative clauses with a singular head and plural attractor—the configuration most susceptible to attraction effects—and contained either a singular or plural target verb. The attraction effect induced in a plural attractor-plural verb configuration reduces the ability to detect ungrammaticality. Thus, performance differences between presentation formats would not tease out individual effects of grammaticality and attraction potential on agreement, but would indicate modulation of the general processing operations that govern this effect at baseline.

The presentation of sentences in a slow word-by-word format has the potential to interfere with the projection of prosody onto the text (Castelhano and Muter, 2001; Fernández, 2007), and thus disrupt processing. Conversely, the segmentation of sentences into appropriate phrasal units may significantly improve performance (O’Shea and Sindelar, 1983; Schreiber, 1987, 1991), easing the processing of grammatical features or aspects by aligning with syntactic structure. It may improve subject-verb agreement processing and possibly improve comprehension for probes targeting relative clause interpretation since a correct response requires parsing of the structural relation between the two relevant nouns.

For native speakers, the clause typically operates as a planning unit in speech, and so pauses tend to occur at clause boundaries (Butterworth, 1980; Garrett, 1982; McDaniel et al., 2010). In contrast, for lower-fluency L2 learners, there is no evidence of the clause as a planning unit: pauses are distributed across the utterance, and there is less hesitation at clause boundaries than in native speech (Temple, 2000). However, as the development of fluent L2 speech progresses, pauses at clausal boundaries increase, and begin to converge with native speech patterns. Thus, even though a certain level of L2 fluency must be attained before native-like pausing patterns emerge, prosodic, and syntactic phrase alignment is a feature of both L1 and L2 processing. If implicit prosody is the projection of prosodic contours onto text, it would follow that similar fluency effects may be found for reading as well.

Because we assume that cue-based retrieval operates independently of language background, L1 and L2 participants’ performance was predicted to be qualitatively similar across tasks. However, because L2 participants may be under higher task demand in all conditions, we predicted that they may show evidence of greater variation based on presentation format.

### Materials and Methods

#### Participants

*L1 participants*: 63 native English speakers were recruited (*mean* 21.6 years, *SD* 6.2 years; 37 female). All were Queens College students enrolled in an introductory psychology course and received course credit for their participation.

*L2 participants:* 24 Spanish–English bilinguals (L2) were recruited (*mean* 23.1 years, *SD* 5.8 years; *mean AoA*: 7.97 years; 15 female). Eighteen were Queens College students enrolled in an introductory psychology course and received course credit for their participation. Six were recruited via flyer or word of mouth, and were compensated for their time. L2 participants were selected based on information provided in a background questionnaire. Inclusion criteria consisted of indication of Spanish as L1 and age of arrival (age of first exposure to an English-dominant environment) as 5 years old or older. Eight additional participants completed the study, but were excluded due to significant data loss from software error (*n* = 6), or failure to follow instructions (*n* = 2).

Average proficiency measures for L1 and L2 participants are given in **Table 2**. Self-assessed proficiency measures were collected using a Language Experience and Proficiency Questionnaire (LEAP-Q) (Marian et al., 2007). All participants were also administered the passage comprehension subtest of the Woodcock-Johnson III Tests of Achievement (Woodcock et al., 2001), which requires silent reading of a short passage and verbal identification of a missing word appropriate to the context. L2 participants were also administered the equivalent passage comprehension subtest of the Batería III Woodcock-Muñoz Pruebas de Aprovechamiento (Woodcock et al., 2004).

**Table 2:** Average proficiency measures for L1 and L2 participants: self-assessments from the LEAP-Q Questionnaire and passage comprehension scores from the Woodcock-Johnson (in English) and Woodcock-Muñoz (in Spanish) batteries.

	L1	L2	
Measure	In English	In English	In Spanish
Self-Assessed Speaking	9.28/10	8.66/10	8.51/10
Self-Assessed Comprehension	9.41/10	8.86/10	8.95/10
Self-Assessed Reading	9.25/10	8.82/10	8.04/10
Passage Comprehension	39.3/47	38.6/47	38.1/47

#### Materials

Experimental materials consisted of subject relative clause sentence sets containing a singular head and an intervening plural attractor noun and either a singular or plural target verb. A sample set of the experimental materials appears in **Table 3** and the full set of materials is available in Supplementary Data Sheet 1. The nouns and target verb were selected from a list of the 5000 most frequent words of each type in the Corpus of Contemporary American English (COCA), and all head and attractor nouns were animate. The 2×2 design crossed the factors of structural complexity (Simple, Complex) and grammaticality (Grammatical, Ungrammatical). Sentences within each set were matched for syllable length by either including an adjunct modifier for the simple sentences, or embedding an additional relative clause for the complex sentences. The complex sentences were constructed using proper nouns, based on evidence that proper nouns are less likely to interfere with agreement computation of common nouns^2^ (possibly due to less feature overlap that would contribute to retrieval interference, see Gordon et al., 2001, 2004; Van Dyke and McElree, 2006). Grammaticality was manipulated by varying whether the number feature of the main verb agreed with the singular subject. Fillers contained agreement violations within other configurations such as noun complements, *wh*-phrases, or pronouns, or violations relating to mass-count number or argument structure.

**Table 3:** Sample set of materials.

Complexity	Grammaticality	Sentence
Simple	Grammatical	(a) The reporter who called the senators every so often writes awful stories for the newspaper.
	Ungrammatical	(b) ^∗^The reporter who called the senators every so often write awful stories for the newspaper.
Complex	Grammatical	(c) The reporter who called the senators that Scott supported writes awful stories for the newspaper.
	Ungrammatical	(d) ^∗^The reporter who called the senators that Scott supported write awful stories for the newspaper.

*^∗^indicates ungrammaticality.*

Experimental items (*n* = 64) were distributed across four lists in a Latin Square design and presented following 16 practice items and interspersed among 128 fillers. Experimental and filler materials were pseudorandomized into four blocks of 48 sentences each. Half of all experimental items were ungrammatical.

#### Procedure

The three presentation paradigms had a unique presentation format and pacing, as detailed below. All three paradigms allowed incorporation of grammaticality ratings and sentence comprehension probes.

In the SENTENCE presentation paradigm, sentences were presented individually on one line in their entirety, and reading was self-paced. Each sentence was preceded by a fixation cross appearing centrally on the screen for 1000 ms. After 7000 ms, a prompt to “respond quickly” appeared in the upper left corner of the display. A final timeout was set at 15000 ms from initial onset. Both the sentence and prompt remained on the screen until the timeout, or until the participant pressed the space bar to proceed to the next screen.

In the WORD presentation paradigm, sentences were presented word-by-word, in rapid serial visual presentation (RSVP) format, at a fixed rate of 500 ms per word.

In the PHRASE presentation paradigm, each sentence was presented in three segments, with phrasal breaks after the head noun, and again after the relative clause, as shown in (4), and reading was self-paced.

(4) (a) Simple: The reporter | who called the senators every so often | write(s)…
     (b) Complex: The reporter | who called the senators that Scott supported | write(s)…

Each sentence was preceded by a fixation cross appearing centrally on the screen for 1000 ms. The first segment of the sentence then appeared centrally on the screen, and after 2000 ms, a prompt to respond quickly appeared in the upper left corner of the display. A final timeout was set at 5000 ms from initial onset. Both the segment and prompt remained on the screen until the timeout, or until the participant pressed the space bar to advance to the next segment.

For all three presentation paradigms, following each sentence, participants were prompted to rate the sentence on a 6-point Likert scale (1 = “very bad,” 6 = “perfect”). To minimize low ratings due to general dislike for these types of structures, participants were instructed to rate each sentence based on whether a 300-level English professor would consider it grammatical. Participants were then prompted to respond to a true/false comprehension probe, and received speed and accuracy feedback on their responses. The comprehension probes for the experimental items targeted either the relative clause interpretation (5a), or general comprehension (5b).

(5) The reporter who called the senators every once in a while writes awful stories for the newspaper.
     (a) *Relative Clause target:* The reporter called the senators.
     (b) *Other target:* The reporter works in television.

Participants were instructed to respond as quickly and accurately as possible, and were allowed 5000 ms to rate the sentence, and an additional 5000 ms to respond to the comprehension probe.

Stimuli were presented on a PC using E-Prime 2.0 software (Psychology Software Tools, Pittsburgh, PA, USA). Each participant was pseudorandomly assigned to a presentation paradigm and stimulus list upon recruitment. Following the main experimental session, participants completed the Language Experience and Proficiency Questionnaire and the Woodcock-Johnson and/or Woodcock-Muñoz passage comprehension subtests.

## Results

Grammaticality ratings and comprehension accuracy data were analyzed, as well as response times for both measures. Statistics are presented as the results of linear mixed-effects models with the maximal random-effects structures justified by the models, calculated over grammaticality ratings, comprehension question accuracy, and response times. Models were fit using R software (version 3.1.3, R Core Team, 2015) and the *lme4* and *lmerTest* packages.

For the comprehension response time (RT) measures, only items for which the comprehension probe was answered correctly were included in the analysis^3^. A response timeout was set at 5000 ms for all measures and response times of less than 250 ms were excluded. The combined data loss resulting from upper and lower cutoffs was less than 5%. All remaining response times that exceeded a threshold of ±2 standard deviations were replaced by the cutoff value (equal to 2 standard deviations beyond the mean), and subsequently log-transformed prior to analysis.

### Grammaticality Ratings

**FIGURE 1:**
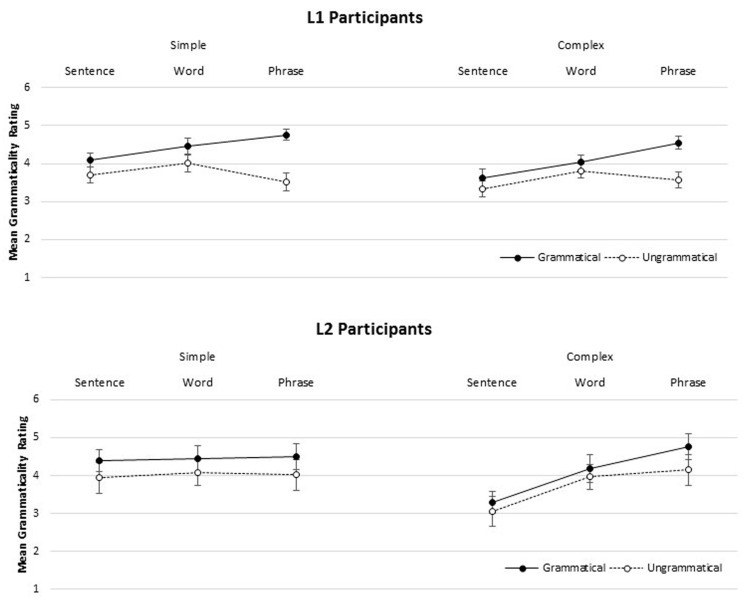
**Mean grammaticality ratings (1 = “very bad,” 6 = “perfect”) for participant groups (L1, Top; L2, Bottom) for simple and complex materials, as a function of presentation format (SENTENCE, WORD, PHRASE).** Error bars are participant-based standard errors.

A linear mixed-effects model was applied to the grammaticality ratings data, and included fixed effects of format (SENTENCE, WORD, PHRASE), participant group (L1, L2), grammaticality (Grammatical, Ungrammatical), and complexity (Simple, Complex), as well as all interactions^4^. The maximal random effects structure justified by the model was used, and from the maximal model, we removed the most complex slopes accounting for the least variance until convergence was reached (Barr et al., 2013). The results of this model, and information on the random-effects structure appear in **Table 4**; summary values for grammaticality ratings appear in **Figure 1**. There was an overall effect of grammaticality: grammatical sentences (*mean* 4.26, *SD* 1.44) were rated higher than ungrammatical sentences, (*mean* 3.76, *SD* 1.51), as well as an overall effect of complexity: simple constructions (*mean* 4.16, *SD* 1.48) were rated higher than complex constructions (*mean* 3.86, *SD* 1.51). Complexity interacted with presentation format, indicating that participants rated complex items higher when presented in the PHRASE format (*mean* 4.26, *SD* 1.53) than in the SENTENCE format (*mean* 3.33, *SD* 1.50).

**Table 4:** Linear mixed-effects model of overall grammaticality ratings.

Fixed Effects	Estimate	*SE*	*df*	*t*	*p*-value	Significance
(Intercept)	4.026	0.11	100	37.76	<0.001	^∗^
Sentence vs. Word	0.190	0.29	89	0.65	0.515	
Sentence vs. Phrase	0.416	0.30	96	1.40	0.165	
Group	-0.119	0.21	92	-0.58	0.566	
Grammaticality	-0.264	0.05	92	-5.41	<0.001	^∗^
Complexity	-0.147	0.03	80	-5.00	<0.001	^∗^
Sentence vs. Word × Group	0.062	0.58	89	0.11	0.915	
Sentence vs. Phrase × Group	-0.291	0.59	95	-0.49	0.624	
Sentence vs. Word × Grammaticality	0.221	0.13	83	1.66	0.101	
Sentence vs. Phrase × Grammaticality	-0.301	0.14	91	-2.18	<0.05	^∗^
Sentence vs. Word × Complexity	0.039	0.07	80	0.57	0.570	
Sentence vs. Phrase × Complexity	0.360	0.07	81	5.20	<0.001	^∗^
Group × Grammaticality	-0.068	0.10	86	-0.71	0.476	
Group × Complexity	0.031	0.05	82	0.64	0.525	
Grammaticality × Complexity	0.038	0.02	4952	1.79	0.074	
Sentence vs. Word × Group × Grammaticality	0.105	0.27	82	0.39	0.695	
Sentence vs. Phrase × Group × Grammaticality	-0.413	0.27	84	-1.54	0.127	
Sentence vs. Word × Group × Complexity	-0.179	0.14	79	-1.30	0.196	
Sentence vs. Phrase × Group × Complexity	-0.328	0.14	80	-2.38	<0.05	^∗^
Sentence vs. Word × Grammaticality × Complexity	0.029	0.06	4864	0.49	0.621	
Sentence vs. Phrase × Grammaticality × Complexity	-0.033	0.06	4967	-0.56	0.574	
Group × Grammaticality × Complexity	0.030	0.05	4727	0.71	0.481	
Sentence vs. Word × Group × Grammaticality × Complexity	-0.033	0.12	4912	-0.29	0.774	
Sentence vs. Phrase × Group × Grammaticality × Complexity	0.156	0.12	5003	1.34	0.181	

*Effects of format, group, grammaticality, and complexity. Sentence vs. Word = Comparison of SENTENCE to WORD, Sentence vs. Phrase = Comparison of SENTENCE to PHRASE; Random effects structure included intercept and effects of Grammaticality and Complexity for Subjects plus intercept and effects of Format, Group, Grammaticality, and Complexity for Items. R-code used with lmerTest: Rating ∼ Format ^∗^ Group ^∗^ Grammaticality ^∗^ Complexity + (1 + Grammaticality + Complexity | Subject) + (1 + Format ^∗^ Group + Format ^∗^ Grammaticality + Complexity | Item). Degrees of freedom (df) calculated using the Satterthwaite approximation. ^∗^indicates a significant result*.

A three-way interaction between group, format, and complexity was investigated further by conducting additional analyses on the L1 and L2 groups separately. Both the L1 and L2 models included fixed effects of format, grammaticality, complexity, and English passage comprehension proficiency from the Woodcock-Johnson battery (WJ Proficiency), as well as all interactions.

Within the L1 group, there was a significant interaction between format and complexity (*Estimate* = 0.181, *SE* = 0.06, *df* = 62, *t* = 2.85, *p* < 0.01), such that ratings were higher for complex items in the PHRASE condition than in the SENTENCE condition. While both groups rated complex items higher in the PHRASE condition, only the L1 group reliably distinguished grammatical from ungrammatical sentences overall, and most robustly in the PHRASE condition (compare the top and bottom panels of **Figure 1**). There was a significant interaction between grammaticality and WJ proficiency, indicating that L1 participants with higher English reading proficiency were better able to correctly accept grammatical sentences (*Estimate* = -0.051, *SE* = 0.02, *df* = 64, *t* = -2.97, *p* < 0.01). For the L2 participants, a format and WJ proficiency interaction revealed that higher WJ proficiency was associated with higher grammaticality ratings in the WORD condition only (*Estimate* = 0.384, *SE* = 0.12, *df* = 24.14, *t* = 3.171, *p* < 0.01). No other results were significant.

Grammaticality ratings for the L2 participants were again analyzed as above, using Spanish passage comprehension proficiency from the Woodcock-Muñoz battery (WM proficiency) as a factor. There was a marginally significant interaction of grammaticality and WM proficiency (*Estimate* = 0.036, *SE* = 0.02, *df* = 20.2, *t* = 1.854, *p* = 0.078), suggesting that higher Spanish reading proficiency may improve ability to correctly accept the grammatical sentences. A significant three-way interaction between format, grammaticality, and WM proficiency (*Estimate* = 0.160, *SE* = 0.06, *df* = 20, *t* = 2.489, *p* < 0.05), indicated that higher proficiency may be particularly beneficial in the PHRASE presentation condition.

### Comprehension Accuracy

**FIGURE 2:**
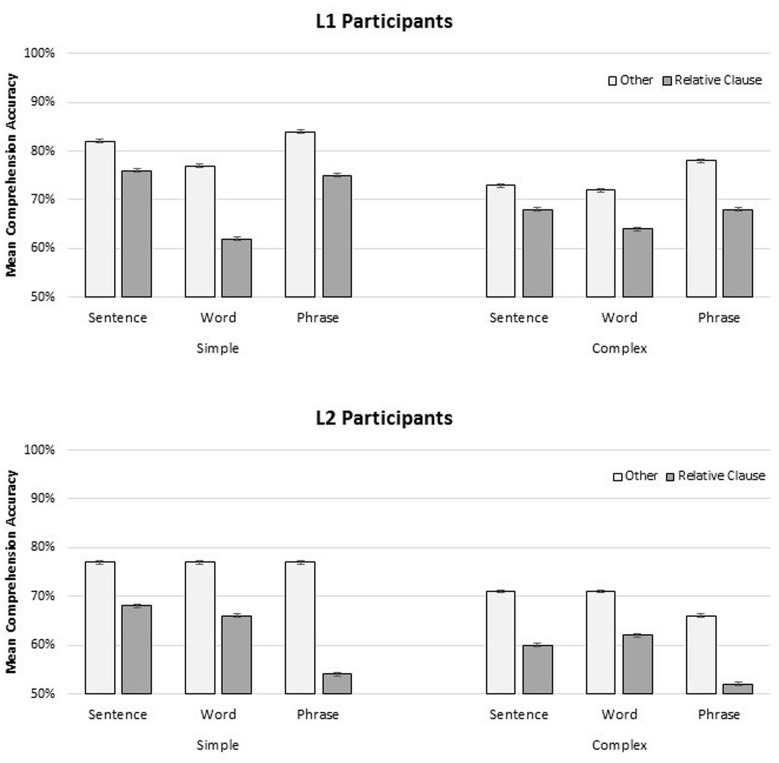
**Mean comprehension accuracy (% correct) for participant groups (L1, **Top**; L2, **Bottom**) for simple and complex materials, for each presentation format (SENTENCE, WORD, PHRASE) as a function of comprehension target (Relative Clause, Other).** Error bars are participant-based standard errors.

A logistic mixed-effects model was applied to the binomial comprehension accuracy data, and included fixed effects of format, group, complexity, and comprehension target (Relative Clause, Other), as well as all interactions. The maximal random effects structure justified by the model was used, and from the maximal model, we removed the most complex slopes accounting for the least variance until convergence was reached. The results of this model, and information on the random-effects structure appear in **Table 5**; summary values for comprehension accuracy are presented in **Figure 2**. Overall comprehension accuracy was higher for L1 participants (*mean* 0.73, *SD* 0.44) than L2 participants (*mean* 0.67, *SD* 0.47) and accuracy was higher for simple constructions (*mean* 0.73, *SD* 0.43) than for complex constructions (*mean* 0.67, *SD* 0.47). There was a significant interaction between format and group (*Estimate* = -0.798, *SE* = 0.33, *z* = -2.444, *p* < 0.05), where accuracy for L1 participants was similar between the SENTENCE (*mean* 0.75, *SD* 0.43) and PHRASE (*mean* 0.76, *SD* 0.43) conditions, but lower in the WORD condition (*mean* 0.69, *SD* 0.42), while accuracy for L2 participants was similar between the SENTENCE (*mean*0.69, *SD* 0.47) and WORD (*mean* 0.69, *SD* 0.46) conditions, but lower in the PHRASE condition (*mean* 0.62, *SD* 0.49) (*Estimate* = 0.863, *SE* = 0.34, *z* = 2.550, *p* < 0.05). The effect of target was also significant: accuracy was lower when the probe targeted the relative clause interpretation (*mean* 0.65, *SD* 0.47) versus general comprehension (*mean* 0.75, *SD* 0.42) (*Estimate*: -0.347, *SE* = 0.14, *z* = -2.532, *p* < 0.05).

**Table 5:** Logistic mixed-effects model of overall comprehension accuracy.

Fixed Effects	Estimate	*SE*	*z*	*p*-value	Significance
(Intercept)	1.117	0.14	7.855	<0.001	^∗^
Sentence vs. Word	-0.178	0.17	-1.071	0.284	
Sentence vs. Phrase	-0.104	0.17	-0.630	0.529	
Group	0.422	0.12	3.628	<0.001	^∗^
Complexity	-0.202	0.06	-3.206	<0.01	^∗^
Target	-0.347	0.14	-2.532	<0.05	^∗^
Sentence vs. Word × Group	-0.798	0.33	-2.444	<0.05	^∗^
Sentence vs. Phrase × Group	0.863	0.34	2.550	<0.05	^∗^
Sentence vs. Word × Complexity	0.147	0.14	1.025	0.305	
Sentence vs. Phrase × Complexity	-0.157	0.14	-1.090	0.276	
Sentence vs. Word × Target	0.052	0.12	-0.380	0.704	
Sentence vs. Phrase × Target	-0.190	0.13	0.413	0.680	
Group × Complexity	-0.038	0.10	-1.507	0.132	
Group × Target	0.055	0.09	0.616	0.538	
Complexity × Target	0.097	0.06	1.689	0.091	
Sentence vs. Word × Group × Complexity	0.031	0.30	0.103	0.918	
Sentence vs. Phrase × Group × Complexity	-0.014	0.29	-0.047	0.962	
Sentence vs. Word × Group × Target	-0.221	0.25	-0.895	0.371	
Sentence vs. Phrase × Group × Target	0.113	0.26	0.429	0.668	
Sentence vs. Word × Complexity × Target	0.033	0.12	0.268	0.789	
Sentence vs. Phrase × Complexity × Target	0.011	0.13	0.089	0.929	
Group × Complexity × Target	-0.007	0.09	-0.073	0.942	
Sentence vs. Word × Group × Complexity × Target	0.119	0.27	0.436	0.663	
Sentence vs. Phrase × Group × Complexity × Target	-0.141	0.25	-0.564	0.573	

*Effects of format, group, complexity, and target. Sentence v. Word = Comparison of SENTENCE to WORD, Sentence v. Phrase = Comparison of SENTENCE to PHRASE; Random effects structure included intercept and effects of Complexity and Target for Subjects plus intercept and effects of Format, Group, and Complexity for Items. R-code used with glmer: Comprehension Accuracy ∼ Format ^∗^ Group ^∗^ Complexity ^∗^ Target + (1 + Complexity ^∗^ Target | Subject) + (1 + Format ^∗^ Group ^∗^ Complexity | Item). ^∗^indicates a significant result.*

### Response Times

The linear mixed-effects model for grammaticality rating RTs included fixed effects of format, group, grammaticality, and complexity, as well as all interactions. Significant findings included a main effect of format (*Estimate* = 0.372, *SE* = 0.12, *df* = 86, *t* = 3.15, *p* < 0.01), as well as an interaction of format and complexity (*Estimate* = 0.038, *SE* = 0.02, *df* = 138, *t* = 2.39, *p* < 0.05), such that response times were slower in the WORD condition, and particularly so for complex constructions.

The linear mixed-effects model for comprehension probe RTs included fixed effects of format, group, complexity, and comprehension target, as well as all interactions. Overall, L2 participants were faster than L1 participants in responding to the comprehension probe (*Estimate* = 0.127, *SE* = 0.05, *df* = 87, *t* = 2.75, *p* < 0.01). Response times were also faster for simple constructions than for complex constructions (*Estimate* = 0.017, *SE* = 0.01, *df* = 77, *t* = 2.53, *p* < 0.05). While overall response times were only marginally faster in the PHRASE condition (*Estimate* = -0.117, *SE* = 0.07, *df* = 88, *t* = -1.79, *p* < 0.08), there was a significant interaction between format and group (*Estimate* = -0.030, *SE* = 0.01, *df* = 87, *t* = -2.30, *p* < 0.05), such that L2 participants responded more quickly to the comprehension probe in the PHRASE condition only.

### Summary of Results

In the SENTENCE format condition, all participants had difficulty appropriately distinguishing grammatical from ungrammatical constructions. However, L1 participants *were* able to reliably distinguish grammatical and ungrammatical items presented in the PHRASE format. The PHRASE format also made the typically dispreferred complex constructions more acceptable for both groups, resulting in higher ratings overall.

Comprehension accuracy was higher for simple than complex items, and higher when the probes targeted general comprehension, rather than relative clause interpretation. Comprehension accuracy was higher overall for L1 participants than L2 participants; however, the groups were differently affected by presentation format. L1 participants were significantly disrupted in the WORD presentation condition, particularly when the comprehension probe targeted the relative clause interpretation. On the other hand, L2 participants were significantly disrupted in the PHRASE presentation condition, again, particularly when the comprehension probe targeted the relative clause.

## Discussion

In this investigation, our overarching goal was to explore how text presentation format interacts with comprehension and agreement processing and to motivate the integration of (implicit) prosodic effects into a cue-based memory retrieval model. Following previous studies, we used subject-verb agreement licensing as a measure of online grammatical processing during reading. While relative clauses are typically considered less susceptible to agreement attraction effects, items were configured to maximize the potential for agreement attraction (singular subject-plural attractor), and vary structural complexity while minimizing length differences that would affect the time course of cue decay.

We have proposed that one of the functions of prosody is to facilitate processing and reduce task demand. This may be partially due to the relation between syntactic and prosodic structure, and partially as a memory aid—by way of prosodic contour, and/or by creating phrasal ‘edges,’ which may strengthen the ability to retrieve elements associated with these edges. Applying this approach to the current design, we anticipated that attraction effects would be stronger (i) in complex items versus simple items, and (ii) in L2 participants versus L1 participants. Regarding the first prediction, looking solely at activation and cue decay across structures, we anticipated higher probability of erroneous retrieval (i.e., higher grammaticality ratings) in complex items than in simple items.

Overall, neither L1 nor L2 participants were able to reliably differentiate grammatical constructions in the SENTENCE condition, regardless of item complexity. However, in the PHRASE condition, the L1 participants were able to discriminate between grammatical and ungrammatical items in the simple sentences, but less so in the complex sentences.

Following previous studies, including the pilot study reported above, the inability in the SENTENCE condition to distinguish between grammatical and ungrammatical constructions likely reflects uncertainty about which NP is the correct controller, due to attraction effects. If this is the case, then the PHRASE condition allowed the L1 participants to respond with more certainty to the grammatical items—particularly in the simple condition—because either (i) alignment of segments with syntactic constituents directly affects retrieval, either by strengthening the cue(s) for the correct controller, reducing interference, or slowing cue decay, (ii) phrasal segmentation otherwise assists memory, mitigating the drain on cognitive resources to allow retrieval to proceed as usual, or (iii) some combination of these two effects.

The data also align with the second prediction that L2 participants may be more susceptible to attraction effects. Not only were the L2 participants unable to differentiate grammatical from ungrammatical items in the SENTENCE condition, they were unable to differentiate the two in any of the presentation conditions. If the L2 participants were more susceptible to retrieval interference, due to processing load or a similar factor, the rate of cue decay prior to onset of the target verb may have hindered correct retrieval, particularly as complexity and subject-verb distance increased.

Neither the L1 nor L2 participants were able to consistently distinguish grammatical from ungrammatical items in the SENTENCE condition, which may be partially due to an attraction effect, i.e., the illusion of grammaticality based on the presence of a plural intervener. However, even ratings for the grammatical experimental items were low (*mean* 3.85, *SD* 1.48) in contrast with ratings for grammatical filler items (*mean* 4.70, *SD* 1.22), suggesting that these constructions may be generally dispreferred due to complexity, independent of grammaticality. This dispreference may relate to proficiency, as seen within the L1 participant group, where higher English reading proficiency (as indicated by Woodcock-Johnson passage comprehension scores) was associated with higher ratings. At higher proficiency levels, readers may be better able to parse complex materials, and thus are less likely to reject them outright.

The comprehension effects seen in both groups reinforce the proposal that presentation format does indeed affect processing during reading. The variation in whether a presentation format facilitates or interferes with comprehension suggests potential differences in how L1 and L2 speakers integrate prosodic features during processing.

While in early L2 development, prosodic phrasing patterns do not initially align with those of native speakers (despite crosslinguistic similarities), appropriate use of prosodic phrasing may develop concurrently with other aspects associated with L2 fluency. This is supported by the findings of Liljestrand Fultz (2009), which suggest that while L2 learners are able to consistently use prosodic information to disambiguate simpler structures (e.g., conjunct modification and PP-attachment constructions), integrating prosodic cues while parsing more complex structures such as relative clauses is difficult, particularly at earlier stages of proficiency. As proficiency increases and processing routines develop, the parser is able to more efficiently integrate information from multiple cues, even when the computation is complex.

Interpreting our results with this view, it may be the case that the L2 participants are generally able to perceive and utilize prosodic cues appropriately. However, when complex computations are required, such as for relative clauses (and particularly in the complex items), those cues along with others, may not be effectively integrated at the point of interpretation, and may also be more subject to interference effects. Future elaboration is needed to determine whether similar effects could be found in oral reading, and with auditory stimuli as well. If so, we can establish the role of prosodic effects with greater certainty, and shed light on the development of prosodic phrasing patterns in L2 speakers.

### The Role of Prosody during Reading

The current results substantiate and consolidate the findings of previous studies regarding the interaction of text segmentation, prosody, and processing. From the data, we see conclusively that text segmentation does indeed influence processing: facilitating agreement computation in the L1 participants, and either facilitating or disrupting comprehension processing in both L1 and L2 participants.

The performance of the L1 participants in the PHRASE condition suggests that phrasal presentation of text reduces processing load and interference created by the attraction configuration and item complexity. This increases the ability to distinguish grammatical from ungrammatical items, an effect found for the L1 participants in both the simple and complex constructions.

Overall, comprehension accuracy was higher for L1 participants than for L2 participants, higher for simple items than complex items, and higher for general comprehension probes than relative clause interpretation probes. However, while the WORD presentation format was disruptive for the L1 participants, the PHRASE presentation format was disruptive for the L2 participants, particularly for the complex items, and when the probe targeted the relative clause. These disparities could reflect differences in the nature and extent of interference effects during cue retrieval. They may be broadly construed as processing load effects, but more specifically could be interpreted as retrieval interference effects. As task demand increases, the resources required to maintain and retrieve the correct subject are depleted, and comprehenders may be increasingly susceptible to similarity-based interference in these configurations. However, presentation format interacts with this effect, such that phrasal presentation reduces interference for some (e.g., L1 participants), and increases it for others (e.g., L2 participants). More work will be needed to specify exactly why L1 and L2 groups may demonstrate different patterns of interference and facilitation.

## Conclusion

The data presented here are not only compatible with a cue-based memory retrieval system for agreement, but also provide support for the claims that implicit prosody contributes to parsing and cue retrieval, with varying effects based on processing load and reading fluency. We anticipate future development of a comprehensive processing paradigm that incorporates syntactic and structural foundations, psycholinguistic evidence, and cognitive processing. The development and refinement of such a paradigm will identify areas for future research, with the added potential to develop pedagogical tools to facilitate performance in L1 reading comprehension, and general L2 acquisition.

Evaluating prosodic features as retrieval cues sets the stage for much future research, particularly with regard to number agreement, the specific role of prosody, and individual processing differences and interference effects in L1 and L2. Another track lies in clarifying the role of (implicit) prosody in performance as a cue to structure, a memory scaffold, or combination of the two. A related concern is linking text presentation effects with oral prosody. The correlation between syntactic and prosodic features creates methodological difficulties in isolating their individual effects, and notably in the current study, the presentation formats of the WORD and PHRASE conditions obligatorily result in structural processing differences in addition to implicit prosodic effects. Thus, it is hypothetically possible that that observed differences between these conditions are due to other processing effects induced by the presentation formats themselves, not directly to implicit prosody. However, we consider this unlikely, particularly as our results strongly align with explicit prosody effects found in comprehension and oral production. As comprehension of complex constructions increases when syntactic structure is marked prosodically (Fodor and Nickels, 2011; Fodor, 2013; Schott and Fodor, 2013), phrasal segmentation of text similarly increases comprehension of written stimuli. The most reliable way to determine the validity of implicit prosody is to evaluate the effect of explicit prosody in equivalent settings, thus, a critical step forward would test this advantage with auditory presentation of the stimuli. There is still much to resolve concerning implicit prosody and phrasing, and to what extent it aligns with explicit prosodic cues. However, this and future work may clarify this relationship by evaluating outcomes within an auditory presentation paradigm, as well as refining conditions to disambiguate between prosodic and non-prosodic effects. Additionally, the current study, which has provided support for an effect of presentation format on processing, sets the foundation for future work to clarify *how* it affects processing, and whether conditions such as grammaticality and attraction potential interact differently and independently within that effect, and between L1 and L2 populations.

Finally, the results suggest that taking advantage of the relationship between prosody and processing may provide innovative approaches to improving comprehension and grammatical processing via prosodic training. Thus, beyond the immediate relevancy of this work to sentence processing research, it has clear application to pedagogical concerns, including the development and testing of interventions for readers with lower fluency and reading comprehension, as well as techniques for presenting text to learners in a facilitative way.

The research presented here has provided insight into the online processing of complex sentences in both L1 and L2 speakers, and demonstrated the importance of prosodic considerations, taskload, and reading fluency in both comprehension and agreement computation. However, much work still remains in clarifying what information is prioritized during parsing, how retrieval and interference effects interact with individual processing profiles, and how this information may be applied productively to developing readers and L2 learners.

## Author Contributions

EP and EF developed the methodology and interpreted the findings. EP designed the overall study, performed the data analysis, and wrote the manuscript.

## Conflict of Interest Statement

The authors declare that the research was conducted in the absence of any commercial or financial relationships that could be construed as a potential conflict of interest.

## Footnotes

1 While cue-based retrieval felicitously explains attraction effects in ungrammatical sentences where both the attractor noun and verb are plural, the patterns of attraction in grammatical sentences (which are much less common, but have been observed) conflict with those predicted by the model. Number agreement may be a special case, such that factors unique to subject-verb number agreement, such as thematic role, focus, and animacy may interact with the retrieval mechanism differently from other dependency relations. See Engelmann et al. (unpublished) for further discussion.

2 Work by Gordon et al. (2001, 2004) suggests that similarity-based interference is reduced or eliminated when common nouns are paired with proper names or pronouns. Further discussion by Van Dyke and McElree (2006) supports this within a cue-based retrieval framework, such that differences in their referential properties would generally preclude a proper name from being mistakenly retrieved as the subject in these configurations. While there is argument for the preferential weighting of syntactic over semantic/discourse features, we extend those assumptions to the materials here, that a proper name in these contexts would be a low-probability match for the target verb.

3 The restriction of RT measures to correct-only responses follows typical practice for many comprehension studies, and was applied here given the overall goal of examining presentation format effects on performance in native and non-native readers. It is important to note that the data from incorrect responses could be equally informative within the context of focused examination of cue-based retrieval in these populations. While beyond the scope of the current discussion, this is a key topic to be revisited in future work.

4 The maximal model, which included English passage comprehension proficiency from the Woodcock-Johnson battery, failed to reach convergence. The measure was excluded from the initial overall analysis, but included in the L1 and L2 analyses.

## References

[B1] Abney S. P. Johnson M. (1991). Memory requirements and local ambiguities of parsing strategies. *J. Psycholinguist. Res.* 20 233–250. 10.1007/BF01067217

[B2] Badecker W. Kuminiak F. (2007). Morphology, agreement and working memory retrieval in sentence production: evidence from gender and case in Slovak. *J. Mem. Lang.* 56 65–85. 10.1016/j.jml.2006.08.004

[B3] Badecker W. Lewis R. L. (2007). “A new theory and computational model of working memory in sentence production: agreement errors as failures of cue-based retrieval,” in *Paper Presented at the 20th Annual CUNY Sentence Processing Conference* (La Jolla, CA: University of California at San Diego).

[B4] Barr D. J. Levy R. Scheepers C. Tily H. J. (2013). Random effects structure for confirmatory hypothesis testing: keep it maximal. *J. Mem. Lang.* 68 255–278. 10.1016/j.jml.2012.11.001 PMC3881361 24403724

[B5] Bock K. Cutting J. C. (1992). Regulating mental energy: performance units in language production. *J. Mem. Lang.* 31 99–127. 10.1016/0749-596X(92)90007-K

[B6] Bock K. Middleton E. L. (2011). Reaching agreement. *Nat. Lang. Linguist. Theory* 29 1033–1069. 10.1007/s11049-011-9148-y

[B7] Bock K. Miller C. A. (1991). Broken agreement. *Cogn. Psychol.* 23 45–93. 10.1016/0010-0285(91)90003-7 2001615

[B8] Butterworth B. (1980). “Evidence from pauses in speech,” in *Language Production: Speech and Talk*, ed. Butterworth B. (London: Academic Press), 155–176.

[B9] Castelhano M. S. Muter P. (2001). Optimizing the reading of electronic text using rapid serial visual presentation. *Behav. Inform. Technol.* 20 237–247. 10.1080/01449290110069400

[B10] Chomsky N. Miller G. A. (1963). “Introduction to the formal analysis of natural languages,” in *Handbook of Mathematical Psychology* Vol. 2 eds Duncan Luce R. Bush R. R. Galanter E. (New York, NY: Wiley), 269–321.

[B11] Clay M. M. Imlach R. H. (1971). Juncture, pitch, and stress as reading behavior variables. *J. Verbal Learn. Verbal Behav.* 10 133–139. 10.1016/S0022-5371(71)80004-X

[B12] Clifton C. Jr. Frazier L. Deevy P. (1999). Feature manipulation in sentence comprehension. *Riv. Linguist.* 11 11–39.

[B13] Cromer W. (1970). The difference model: a new explanation for some reading difficulties. *J. Educ. Psychol.* 61 471–483. 10.1037/h0030288 5483823

[B14] Cutler A. (2012). *Native Listening: Language Experience and the Recognition of Spoken Words.* Cambridge, MA: MIT Press.

[B15] Dekydtspotter L. Donaldson B. Edmonds A. C. Fultz A. L. Petrush R. A. (2008). Syntactic and prosodic computations in the resolution of relative clause attachment ambiguity by English-French learners. *Stud. Second Lang. Acquis.* 30 453 10.1017/S0272263108080728

[B16] Dowhower S. L. (1987). Effects of repeated reading on second-grade transitional readers’ fluency and comprehension. *Read. Res. Q.* 22 389–406. 10.2307/747699

[B17] Eberhard K. M. (1999). The accessibility of conceptual number to the processes of subject–verb agreement in English. *J. Mem. Lang.* 41 560–578. 10.1006/jmla.1999.2662

[B18] Eberhard K. M. Cutting J. C. Bock K. (2005). Making syntax of sense: number agreement in sentence production. *Psychol. Rev.* 112 531–559. 10.1037/0033-295X.112.3.531 16060750

[B19] Fernández E. M. (2007). “How might a rapid serial visual presentation of text affect the prosody projected implicitly during silent reading?,” in *Proceedings of the Conferências do V Congresso Internacional da Associaçao Brasiliera de Lingüistica* Vol. 5 Belo Horizonte, 117–154.

[B20] Fodor J. D. (1998). Learning to parse? *J. Psycholinguist. Res.* 27 285–319. 10.1023/A:1023258301588

[B21] Fodor J. D. (2002). “Psycholinguistics cannot escape prosody,” in *Proceedings of the 1st International Conference on Speech Prosody*, Aix-en-Provence, 83–88.

[B22] Fodor J. D. (2013). “Pronouncing and comprehending center-embedded sentences,” in *Language Down the Garden Path: The Cognitive and Biological Basis for Linguistic Structures*, eds Sanz M. Laka I. Tanenhaus M. K. (Oxford: Oxford University Press).

[B23] Fodor J. D. Nickels S. (2011). “Center-embedded sentences: phrase length, prosody and comprehension,” in *Poster presented at AMLaP 2011*, Paris.

[B24] Foote R. (2011). Integrated knowledge of agreement in early and late English–Spanish bilinguals. *Appl. Psycholinguist.* 32 187–220. 10.1017/S0142716410000342

[B25] Franck J. (2011). Reaching agreement as a core syntactic process: commentary of Bock & Middleton “Reaching Agreement.” *Nat. Lang. Linguist. Theory* 29 1071–1086. 10.1007/s11049-011-9153-1

[B26] Franck J. Vigliocco G. Antón-Méndez I. Collina S. Frauenfelder U. H. (2008). The interplay of syntax and form in sentence production: a cross-linguistic study of form effects on agreement. *Lang. Cogn. Process.* 23 329–374. 10.1080/01690960701467993

[B27] Franck J. Vigliocco G. Nicol J. (2002). Subject-verb agreement errors in French & English: the role of syntactic hierarchy. *Lang. Cogn. Process.* 17 371–404. 10.1080/01690960143000254

[B28] Franck J. Wagers M. W. (2015). “Hierarchical structure and memory retrieval mechanisms in attraction: an SAT study,” in *Paper Presented at the 28th Annual CUNY Conference on Human Sentence Processing*, Los Angeles, CA.

[B29] Freedman S. Forster K. (1985). The psychological status of overgenerated sentences. *Cognition* 26 171–186. 10.1016/0010-0277(85)90015-0 4017513

[B30] Fultz A. L. (2009). *Prosody in Lexical and Syntactic Disambiguation in English-French Interlanguage*, Ph.D. thesis, Indiana University, Bloomington, IN.

[B31] Gordon P. C. Hendrick R. Johnson M. (2001). Memory interference during language processing. *J. Exp. Psychol. Learn. Mem. Cogn.* 27 1411–1423. 11713876 10.1037//0278-7393.27.6.1411

[B32] Garrett M. F. (1982). “Production of speech: observations from normal and pathological language use,” in *Normality and Pathology in Cognitive Functions*, ed. Ellis A. W. (London: Academic Press), 19–76.

[B33] Gordon P. C. Hendrick R. Johnson M. (2004). Effects of noun phrase type on sentence complexity. *J. Mem. Lang.* 51 97–114. 10.1016/j.jml.2004.02.003

[B34] Harley B. Howard J. Hart D. (1995). Second language processing at different ages: do younger learners pay more attention to prosodic cues to sentence structure? *Lang. Learn.* 45 43–71. 10.1111/j.1467-1770.1995.tb00962.x

[B35] Häussler J. Bader M. (2009). Agreement checking and number attraction in sentence comprehension: insights from german relative clauses. *Trav. Cercle Linguist. Prague* 7.

[B36] Hirotani M. Frazier L. Rayner K. (2006). Punctuation and intonation effects on clause and sentence wrap-up: evidence from eye movements. *J. Mem. Lang.* 54 425–443. 10.1016/j.cognition.2008.12.011

[B37] Hopp H. (2007). *Ultimate Attainment at the Interfaces in Second Language Acquisition: Grammar and Processing.* Groningen: Center for Language and Cognition.

[B38] Jiang N. (2004). Morphological insensitivity in second language processing. *Appl. Psycholinguist.* 25 603–634. 10.1017/S0142716404001298

[B39] Jiang N. (2007). Selective integration of linguistic knowledge in adult second language learning. *Lang. Learn.* 57 1–33. 10.1111/j.1467-9922.2007.00397.x

[B40] Johns C. L. Matsuki K. Van Dyke J. A. (2015). Poor readers’ retrieval mechanism: efficient access is not dependent on reading skill. *Front. Psychol.* 6:1552 10.3389/fpsyg.2015.01552 PMC4607860 26528212

[B41] Johnson J. S. Shenkman K. D. Newport E. L. Medin D. L. (1996). Indeterminacy in the grammar of adult language learners. *J. Mem. Lang.* 35 335–352. 10.1006/jmla.1996.0019

[B42] Kaan E. (2002). Investigating the effects of distance and number interference in processing subject-verb dependencies: an ERP study. *J. Psycholinguist. Res.* 31 165–193. 10.1023/A:1014978917769 12022794

[B43] Kadota S. (1982). Some psycholinguistic experiments on the process of reading comprehension. *J. Assumption Jr. Coll.* 9 49–70.

[B44] Kadota S. Tada M. (1992). Eibun oyobi nihonbun no dokkai to shoritani [Reading comprehension and processing units in English and Japanese]. *Annu. Bull. Res. Inst. Soc. Sci.* 22 137–153.

[B45] Keating G. (2009). Sensitivity to violations of gender agreement in native and nonnative Spanish. *Lang. Learn.* 59 503–535. 10.1111/j.1467-9922.2009.00516.x

[B46] Kilborn K. (1992). “On-line integration of grammatical information in a second language,” in *Cognitive Processing in Bilinguals*, ed. Harris R. J. (Amsterdam: Elsevier), 337–350.

[B47] Kreiner H. (2005). “The role of reading prosody in syntactic and semantic integration: evidence from eye movements,” in *Proceedings of the International Symposium on Discourse and Prosody as a Complex Interface*, At Aix-en-Provence, 1–17.

[B48] Lago S. Shalom D. E. Sigman M. Lau E. F. Phillips C. (2015). Agreement attraction in Spanish comprehension. *J. Mem. Lang.* 82 133–149. 10.1016/j.bandl.2014.05.001

[B49] Lardiere D. (1998). Dissociating syntax from morphology in a divergent L2 end-state grammar. *Second Lang. Res.* 14 359–375. 10.1191/026765898672500216

[B50] LeVasseur V. M. Macaruso P. Palumbo L. C. Shankweiler D. (2006). Syntactically cued text facilitates oral reading fluency in developing readers. *Appl. Psycholinguist.* 27 423–445. 10.1017/S0142716406060346

[B51] LeVasseur V. M. Macaruso P. Shankweiler D. (2008). Promoting gains in reading fluency: a comparison of three approaches. *Read. Writ.* 21 205–230. 10.1007/s11145-007-9070-1

[B52] Lewis R. L. Vasishth S. (2005). An activation-based model of sentence processing as skilled memory retrieval. *Cogn. Sci.* 29 375–419. 10.1207/s15516709cog0000_25 21702779

[B53] MacWhinney B. (1986). “Toward a psycholinguistically plausible parser,” in *Proceedings of the Eastern States Conference on Linguistics (ESCOL) 1986*, ed. Thomason S. (Columbus, OH: Ohio State University), 1–8.

[B54] Marian V. Blumenfeld H. K. Kaushanskaya M. (2007). Language experience and proficiency questionnaire (LEAP-Q): assessing language profiles in bilinguals and multilinguals. *J. Speech Lang. Hear. Res.* 50 940–967. 10.1044/1092-4388(2007/067) 17675598

[B55] McDaniel D. McKee C. Garrett M. F. (2010). Children’s sentence planning: syntactic correlates of fluency variations. *J. Child Lang.* 37 59–94. 10.1017/S0305000909009507 19523262

[B56] McDonald J. L. (2006). Beyond the critical period: processing-based explanations for poor grammaticality judgment performance by late second language learners. *J. Mem. Lang.* 55 381–401. 10.1016/j.jml.2006.06.006

[B57] McElree B. (2001). Working memory and focal attention. *J. Exp. Psychol. Learn. Mem. Cogn.* 27 817–835. 11394682 PMC3077110

[B58] McElree B. (2006). “Accessing recent events,” in *Psychology of Learning and Motivation: Advances in Research and Theory* Vol. 46 ed. Ross B. H. (San Diego, CA: Academic Press), 155–200.

[B59] McElree B. Foraker S. Dyer L. (2003). Memory structures that subserve sentence comprehension. *J. Mem. Lang.* 48 67–91. 10.1207/s15516709cog0000_25

[B60] Miller G. A. Chomsky N. (1963). “Finitary models of language users,” in *Handbook of mathematical psychology* Vol. 2 eds Luce R. D. Bush R. R. Galanter E. (New York, NY: Wiley), 419–491.

[B61] Nicol J. (1995). Effects of clausal structure on subject-verb agreement errors. *J. Psycholinguist. Res.* 24 507–516. 10.1007/BF02143164 8531170

[B62] Nicol J. Forster K. Veres C. (1997). Subject-verb agreement processes in comprehension. *J. Mem. Lang.* 36 569–587. 10.1006/jmla.1996.2497

[B63] O’Shea L. J. Sindelar P. T. (1983). The effects of segmenting written discourse on the reading comprehension of low- and high-performance readers. *Read. Res. Q.* 18 458–465. 10.2307/747380

[B64] Osterhout L. Mobley L. A. (1995). Event-related brain potentials elicited by failure to agree. *J. Mem. Lang.* 34 739–773. 10.1006/jmla.1995.1033

[B65] Paige D. D. Rasinski T. V. Magpuri-Lavell T. Smith G. S. (2014). Interpreting the relationships among prosody, automaticity, accuracy, and silent reading comprehension in secondary students. *J. Lit. Res.* 46 123–156.

[B66] Pearlmutter N. J. Garnsey S. M. Bock K. (1999). Agreement processes in sentence comprehension. *J. Mem. Lang.* 41 427–456. 10.1006/jmla.1999.2653

[B67] R Core Team (2015). *R: A Language and Environment for Statistical Computing.* Vienna: The R Foundation for Statistical Computing.

[B68] Rasinski T. V. Padak N. Linek W. Sturtevant E. (1994). Effects of fluency development on urban second-grade readers. *J. Educ. Res.* 87 158–165. 10.1080/00220671.1994.9941237

[B69] Rayner K. Pollatsek A. Ashby J. Clifton C. Jr. (2012). *Psychology of Reading*, 2nd Edn. New York, NY: Psychology Press.

[B70] Sato M. Felser C. (2006). Sensitivity to semantic and morphosyntactic violations in L2 sentence processing: evidence from speeded grammaticality judgements. *Essex Rep. Linguist.* 51 1–39.

[B71] Schott E. Fodor J. D. (2013). “Prosody induced by phrase lengths facilitates syntactic processing in reading,” in *Poster Presented at AMLaP 2013*, Marseilles.

[B72] Schreiber P. A. (1987). “Prosody and structure in children’s syntactic processing,” in *Comprehending oral and written language*, eds Horowitz R. Samuels S. J. (New York, NY: Academic Press), 243–270.

[B73] Schreiber P. A. (1991). Understanding prosody’s role in reading acquisition. *Theory Pract.* 30 158–164. 10.1080/00405849109543496

[B74] Solomon E. S. Pearlmutter N. J. (2004). Semantic integration and syntactic planning in language production. *Cogn. Psychol.* 49 1–46. 10.1016/j.cogpsych.2003.10.001 15193971

[B75] Staub A. (2007). The parser doesn’t ignore intransitivity, after all. *J. Exp. Psychol. Learn. Mem. Cogn.* 33 550–569. 17470005 10.1037/0278-7393.33.3.550 PMC4626212

[B76] Tanner D. (2011). *Agreement Mechanisms in Native and Nonnative Language Processing: Electrophysiological Correlates of Complexity and Interference.* Washington, DC: University of Washington.

[B77] Temple L. (2000). Second language learner speech production. *Stud. Linguist.* 54 288–297. 10.1111/1467-9582.00068

[B78] Thornton R. MacDonald M. C. (2003). Plausibility and grammatical agreement. *J. Mem. Lang.* 48 740–759. 10.1016/S0749-596X(03)00003-2

[B79] Van Dyke J. A. Lewis R. L. (2003). Distinguishing effects of structure and decay on attachment and repair: a cue-based parsing account of recovery from misanalyzed ambiguities. *J. Mem. Lang.* 49 285–316. 10.1016/S0749-596X(03)00081-0

[B80] Van Dyke J. A. McElree B. (2006). Retrieval interference in sentence comprehension. *J. Mem. Lang.* 55 157–166. 10.1016/j.jml.2006.03.007 18209744 PMC2206541

[B81] Van Dyke J. A. McElree B. (2011). Cue-dependent interference in comprehension. *J. Mem. Lang.* 65 247–263. 10.1016/j.jml.2011.05.002 21927535 PMC3171743

[B82] Vigliocco G. Butterworth B. Garrett M. F. (1996a). Subject-verb agreement in Spanish and English: differences in the role of conceptual constraints. *Cognition* 61 261–298. 10.1016/S0010-0277(96)00713-5 8990974

[B83] Vigliocco G. Hartsuiker R. J. Jarema G. Kolk H. H. J. (1996b). One or more labels on the bottles? Notional concord in Dutch and French. *Lang. Cogn. Process.* 11 407–442. 10.1080/016909696387169

[B84] Vigliocco G. Nicol J. (1998). Separating hierarchical relations and word order in language production: is proximity concord syntactic or linear? *Cognition* 68 B13–B29. 10.1016/S0010-0277(98)00041-9 9775519

[B85] Wagers M. W. (2008). *The Structure of Memory Meets Memory for Structure in Linguistic Cognition.* College Park, MD: University of Maryland, College Park.

[B86] Wagers M. W. Lau E. F. Phillips C. (2009). Agreement attraction in comprehension: representations and processes. *J. Mem. Lang.* 61 206–237. 10.3389/fpsyg.2015.00349

[B87] Wanner E. Maratsos M. (1978). “An ATN approach to comprehension,” in *Linguistic Theory and Psychological Reality*, eds Halle M. Bresnan J. Miller G. A. (Cambridge, MA: MIT Press), 119–161.

[B88] Woodcock R. W. McGrew K. S. Mather N. (2001). *Woodcock-Johnson III Tests of Achievement.* Itasca, IL: Riverside Publishing.

[B89] Woodcock R. W. Muñoz-Sandoval A. F. McGrew K. S. Mather N. (2004). *Batería III Woodcock-Muñoz Pruebas de Aprovechamiento.* Itasca, IL: Riverside Publishing.

[B90] Yamashita J. Ichikawa S. (2010). Examining reading fluency in a foreign language?: effects of text segmentation on L2 readers. *Read. Foreign Lang.* 22 263–283.

