# Supplementary material for: Implicit Prosody and Cue-based Retrieval: L1 and L2 Agreement and Comprehension during Reading
Source: Front Psychol. 2016 Dec 15;7:1922. doi: 10.3389/fpsyg.2016.01922 (PMC5156782; doi:10.3389/fpsyg.2016.01922)
Supplement: Supplementary file 1 [file Data_Sheet_1.PDF]

Supplementary Material

Implicit prosody and cue-based retrieval: L1 and L2 agreement and comprehension during reading

Elizabeth Pratt 1*, Eva M. Fernández 2

*** Correspondence:** Elizabeth Pratt, epratt@gradcenter.cuny.edu

1 Experimental Items

a. Simple, Grammatical
b. Simple, Ungrammatical
c. Complex, Grammatical
d. Complex, Ungrammatical

1. a. The reporter who called the senators every so often writes awful stories for the newspaper.
   b. The reporter who called the senators every so often write awful stories for the newspaper.
   c. The reporter who called the senators that Scott supported writes awful stories for the newspaper.
   d. The reporter who called the senators that Scott supported write awful stories for the newspaper.
2. a. The chef who disliked the critics from the show has many years of experience in restaurants.
   b. The chef who disliked the critics from the show have many years of experience in restaurants.
   c. The chef who disliked the critics that Jeff sent has many years of experience in restaurants.
   d. The chef who disliked the critics that Jeff sent have many years of experience in restaurants.
3. a. The coach who loved the players on the soccer team encourages everyone before each big game.
   b. The coach who loved the players on the soccer team encourage everyone before each big game.
   c. The coach who loved the players that Billy trained encourages everyone before each big game.
   d. The coach who loved the players that Billy trained encourage everyone before each big game.
4. a. The contractor who paid the inspectors from the office approves the expenses for every project.
   b. The contractor who paid the inspectors from the office approve the expenses for every project.
   c. The contractor who paid the inspectors that Alison hired approves the expenses for every project.
   d. The contractor who paid the inspectors that Alison hired approve the expenses for every project.
5. a. The designer who called the retailers several times a week buys expensive silks for her customers.
   b. The designer who called the retailers several times a week buy expensive silks for her customers.
   c. The designer who called the retailers that Max sponsored buys expensive silks for her customers.
   d. The designer who called the retailers that Max sponsored buy expensive silks for her customers.
6. a. The engineer who brought the suppliers to the warehouse assembles all the models of the equipment.
   b. The engineer who brought the suppliers to the warehouse assemble all the models of the equipment.
   c. The engineer who brought the suppliers that Anita met assembles all the models of the equipment.
   d. The engineer who brought the suppliers that Anita met assemble all the models of the equipment.
7. a. The attorney who interviewed the analysts before the trial prepares every case with special experts.
   b. The attorney who interviewed the analysts before the trial prepare every case with special experts.
   c. The attorney who interviewed the analysts that Lynn requested prepares every case with special experts.
   d. The attorney who interviewed the analysts that Lynn requested prepare every case with special experts.
8. a. The fisherman who questioned the merchants from the market handles the finances of the group.
   b. The fisherman who questioned the merchants from the market handle the finances of the group.
   c. The fisherman who questioned the merchants that Maria used handles the finances of the group.

d. The fisherman who questioned the merchants that Maria used handle the finances of the group.

9. a. The guard who attacked the inmates in the prison yard punishes anyone who steps out of line.
   b. The guard who attacked the inmates in the prison yard punish anyone who steps out of line.
   c. The guard who attacked the inmates that Mitch escorted punishes anyone who steps out of line.
   d. The guard who attacked the inmates that Mitch escorted punish anyone who steps out of line.

10. a. The guide who approached the travelers at the station warns his clients about dangerous areas.
    b. The guide who approached the travelers at the station warn his clients about dangerous areas.
    c. The guide who approached the travelers that Bill left warns his clients about dangerous areas.
    d. The guide who approached the travelers that Bill left warn his clients about dangerous areas.

11. a. The instructor who disliked the observers from Chicago believes that all teenagers are spoiled.
    b. The instructor who disliked the observers from Chicago believe that all teenagers are spoiled.
    c. The instructor who disliked the observers that Isabel sent believes that all teenagers are spoiled.
    d. The instructor who disliked the observers that Isabel sent believe that all teenagers are spoiled.

12. a. The banker who brought the clerks to the boardroom meets with the shareholders every month.
    b. The banker who brought the clerks to the boardroom meet with the shareholders every month.
    c. The banker who brought the clerks that Carrie hired meets with the shareholders every month.
    d. The banker who brought the clerks that Carrie hired meet with the shareholders every month.

13. a. The judge who recognized the attorneys before the trial records every meeting in the courthouse.
    b. The judge who recognized the attorneys before the trial record every meeting in the courthouse.
    c. The judge who recognized the attorneys that Sam paid records every meeting in the courthouse.
    d. The judge who recognized the attorneys that Sam paid record every meeting in the courthouse.

14. a. The manager who approved the associates for a promotion closes the office late every Friday night.
    b. The manager who approved the associates for a promotion close the office late every Friday night.
    c. The manager who approved the associates that Jane hired closes the office late every Friday night.
    d. The manager who approved the associates that Jane hired close the office late every Friday night.

15. a. The manufacturer who cheated the sellers on a shipment evaluates every business deal carefully.
    b. The manufacturer who cheated the sellers on a shipment evaluate every business deal carefully.
    c. The manufacturer who cheated the sellers that Gus referred evaluates every business deal carefully.
    d. The manufacturer who cheated the sellers that Gus referred evaluate every business deal carefully.

16. a. The mechanic who sent the agents to the auto show restores vintage cars for several private buyers.
    b. The mechanic who sent the agents to the auto show restore vintage cars for several private buyers.
    c. The mechanic who sent the agents that Erica knew restores vintage cars for several private buyers.
    d. The mechanic who sent the agents that Erica knew restore vintage cars for several private buyers.

17. a. The owner who discussed the developers at the meeting appreciates honesty in negotiations.
    b. The owner who discussed the developers at the meeting appreciate honesty in negotiations.
    c. The owner who discussed the developers that Pat advised appreciates honesty in negotiations.
    d. The owner who discussed the developers that Pat advised appreciate honesty in negotiations.

18. a. The painter who knew the buyers from the museum arranges every purchase through an accountant.
    b. The painter who knew the buyers from the museum arrange every purchase through an accountant.
    c. The painter who knew the buyers that Jane contacted arranges every purchase through an accountant.
    d. The painter who knew the buyers that Jane contacted arrange every purchase through an accountant.

19. a. The person who replaced the employees in the business office needs two jobs to support the family.
    b. The person who replaced the employees in the business office need two jobs to support the family.
    c. The person who replaced the employees that Paul recommended needs two jobs to support the family.
    d. The person who replaced the employees that Paul recommended need two jobs to support the family.

20. a. The photographer who impressed the journalists at the conference requests new assignments every month.
    b. The photographer who impressed the journalists at the conference request new assignments every month.
    c. The photographer who impressed the journalists that Matthew sent requests new assignments every month.
    d. The photographer who impressed the journalists that Matthew sent request new assignments every month.

21. a. The producer who found the sponsors for the new season promises to make every show a success.

b. The producer who found the sponsors for the new season promise to make every show a success.
c. The producer who found the sponsors that Julie wanted promises to make every show a success.
d. The producer who found the sponsors that Julie wanted promise to make every show a success.

22. a. The resident who respected the nurses in the retirement home uses charm to get favors from the staff.
b. The resident who respected the nurses in the retirement home use charm to get favors from the staff.
c. The resident who respected the nurses that Kim supervised uses charm to get favors from the staff.
d. The resident who respected the nurses that Kim supervised use charm to get favors from the staff.

23. a. The senior who recruited the freshmen at the career fair encourages student involvement in politics.
b. The senior who recruited the freshmen at the career fair encourage student involvement in politics.
c. The senior who recruited the freshmen that Tom advised encourages student involvement in politics.
d. The senior who recruited the freshmen that Tom advised encourage student involvement in politics.

24. a. The specialist who appointed the physicians from the clinic recruits new employees every year.
b. The specialist who appointed the physicians from the clinic recruit new employees every year.
c. The specialist who appointed the physicians that John met recruits new employees every year.
d. The specialist who appointed the physicians that John met recruit new employees every year.

25. a. The spokesman who introduced the experts to the sales team gets nervous in front of an audience.
b. The spokesman who introduced the experts to the sales team get nervous in front of an audience.
c. The spokesman who introduced the experts that Judy called gets nervous in front of an audience.
d. The spokesman who introduced the experts that Judy called get nervous in front of an audience.

26. a. The supervisor who welcomed the aides from the field office motivates others to work diligently.
b. The supervisor who welcomed the aides from the field office motivate others to work diligently.
c. The supervisor who welcomed the aides that Barbara transferred motivates others to work diligently.
d. The supervisor who welcomed the aides that Barbara transferred motivate others to work diligently.

27. a. The author who confronted the publishers about the contract refuses to revise any of her manuscripts.
b. The author who confronted the publishers about the contract refuse to revise any of her manuscripts.
c. The author who confronted the publishers that Jack admired refuses to revise any of her manuscripts.
d. The author who confronted the publishers that Jack admired refuse to revise any of her manuscripts.

28. a. The surgeon who visited the patients after their treatments dislikes traditional medicine and procedures.
b. The surgeon who visited the patients after their treatments dislike traditional medicine and procedures.
c. The surgeon who visited the patients that Lois treated dislikes traditional medicine and procedures.
d. The surgeon who visited the patients that Lois treated dislike traditional medicine and procedures.

29. a. The teacher who noticed the parents in the principal's office endorses discipline in the classroom.
b. The teacher who noticed the parents in the principal's office endorse discipline in the classroom.
c. The teacher who noticed the parents that Marcie greeted endorses discipline in the classroom.
d. The teacher who noticed the parents that Marcie greeted endorse discipline in the classroom.

30. a. The technician who chose the assistants for the renovations arrives every day after five o'clock.
b. The technician who chose the assistants for the renovations arrive every day after five o'clock.
c. The technician who chose the assistants that Caroline hired arrives every day after five o'clock.
d. The technician who chose the assistants that Caroline hired arrive every day after five o'clock.

31. a. The trainer who inspired the athletes at the high school supports community service projects.
b. The trainer who inspired the athletes at the high school support community service projects.
c. The trainer who inspired the athletes that Julie coached supports community service projects.
d. The trainer who inspired the athletes that Julie coached support community service projects.

32. a. The volunteer who trained the assistants in only two weeks works every Monday at the shelter.
b. The volunteer who trained the assistants in only two weeks work every Monday at the shelter.
c. The volunteer who trained the assistants that Diego brought works every Monday at the shelter.
d. The volunteer who trained the assistants that Diego brought work every Monday at the shelter.

33. a. The advisor who quickly picked the defendants from a list keeps legal documents for many clients.
b. The advisor who quickly picked the defendants from a list keep legal documents for many clients.
c. The advisor who picked the defendants that Gary accused keeps legal documents for many clients.
d. The advisor who picked the defendants that Gary accused keep legal documents for many clients.

34. a. The ambassador who happily presented the diplomats to the queen organizes events for all visitors.
    b. The ambassador who happily presented the diplomats to the queen organize events for all visitors.
    c. The ambassador who presented the diplomats that Lauren interviewed organizes events for all visitors.
    d. The ambassador who presented the diplomats that Lauren interviewed organize events for all visitors.

35. a. The candidate who briefly met the senators from Indiana receives secret funding from many banks.
    b. The candidate who briefly met the senators from Indiana receive secret funding from many banks.
    c. The candidate who met the senators that Lee introduced receives secret funding from many banks.
    d. The candidate who met the senators that Lee introduced receive secret funding from many banks.

36. a. The chairman who kindly thanked the directors in a letter donates money to the local shelter.
    b. The chairman who kindly thanked the directors in a letter donate money to the local shelter.
    c. The chairman who thanked the directors that Clark assisted donate money to the local shelter.
    d. The chairman who thanked the directors that Clark assisted donates money to the local shelter.

37. a. The columnist who suddenly attacked the fans in the crowd blames entertainers for corrupting the city.
    b. The columnist who suddenly attacked the fans in the crowd blame entertainers for corrupting the city.
    c. The columnist who attacked the fans that Diane invited blames entertainers for corrupting the city.
    d. The columnist who attacked the fans that Diane invited blame entertainers for corrupting the city.

38. a. The artist who once interviewed the dealers from the gallery wins many awards for her shows.
    b. The artist who once interviewed the dealers from the gallery win many awards for her shows.
    c. The artist who interviewed the dealers that Todd suggested wins many awards for her shows.
    d. The artist who interviewed the dealers that Todd suggested win many awards for her shows.

39. a. The executive who likely intimidated the competitors from China hosts elegant lunches twice a year.
    b. The executive who likely intimidated the competitors from China host elegant lunches twice a year.
    c. The executive who intimidated the competitors that Carl funded hosts elegant lunches twice a year.
    d. The executive who intimidated the competitors that Carl funded host elegant lunches twice a year.

40. a. The consultant who eagerly helped the architects from the firm likes colorful and unique designs.
    b. The consultant who eagerly helped the architects from the firm like colorful and unique designs.
    c. The consultant who helped the architects that Mario hired likes colorful and unique designs.
    d. The consultant who helped the architects that Mario hired like colorful and unique designs.

41. a. The spy who rightly suspected the officers in the Pentagon hides his gear in a hidden closet.
    b. The spy who rightly suspected the officers in the Pentagon hide his gear in a hidden closet.
    c. The spy who suspected the officers that Sean consulted hides his gear in a hidden closet.
    d. The spy who suspected the officers that Sean consulted hide his gear in a hidden closet.

42. a. The counselor who briefly met the survivors of the flood prefers to organize group therapy sessions.
    b. The counselor who briefly met the survivors of the flood prefer to organize group therapy sessions.
    c. The counselor who met the survivors that Kelly rescued prefers to organize group therapy sessions.
    d. The counselor who met the survivors that Kelly rescued prefer to organize group therapy sessions.

43. a. The employee who first reported the vendors to the bureau has many children to support alone.
    b. The employee who first reported the vendors to the bureau have many children to support alone.
    c. The employee who reported the vendors that Fay interviewed has many children to support alone.
    d. The employee who reported the vendors that Fay interviewed have many children to support alone.

44. a. The criminal who greatly admired the detectives on the case feels guilty after committing a crime.
    b. The criminal who greatly admired the detectives on the case feel guilty after committing a crime.
    c. The criminal who admired the detectives that Kate picked feels guilty after committing a crime.
    d. The criminal who admired the detectives that Kate picked feel guilty after committing a crime.

45. a. The politician who now financed the activists from France admits to forging several hundred documents.
    b. The politician who now financed the activists from France admit to forging several hundred documents.
    c. The politician who financed the activists that Bill caught admits to forging several hundred documents.
    d. The politician who financed the activists that Bill caught admit to forging several hundred documents.

46. a. The customer who completely ignored the managers of the store pays for everything using only coins.
    b. The customer who completely ignored the managers of the store pay for everything using only coins.

c. The customer who ignored the managers that Jonathan called pays for everything using only coins.
d. The customer who ignored the managers that Jonathan called pay for everything using only coins.

47. a. The driver who openly insulted the tourists from the city enjoys traveling through the countryside.
b. The driver who openly insulted the tourists from the city enjoy traveling through the countryside.
c. The driver who insulted the tourists that Karen accompanied enjoys traveling through the countryside.
d. The driver who insulted the tourists that Karen accompanied enjoy traveling through the countryside.

48. a. The musician who angrily fired the producers before the show plays several concerts every week.
b. The musician who angrily fired the producers before the show play several concerts every week.
c. The musician who fired the producers that Roger recommended plays several concerts every week.
d. The musician who fired the producers that Roger recommended play several concerts every week.

49. a. The officer who calmly saved the soldiers during the battle is quite popular with the civilians.
b. The officer who calmly saved the soldiers during the battle are quite popular with the civilians.
c. The officer who saved the soldiers that Steven accompanied is quite popular with the civilians.
d. The officer who saved the soldiers that Steven accompanied are quite popular with the civilians.

50. a. The pilot who rudely approached the passengers in first class is always nervous before a flight.
b. The pilot who rudely approached the passengers in first class are always nervous before a flight.
c. The pilot who approached the passengers that Christine brought is always nervous before a flight.
d. The pilot who approached the passengers that Christine brought are always nervous before a flight.

51. a. The teacher who wisely advised the principals at the conference has no interest in teaching music.
b. The teacher who wisely advised the principals at the conference have no interest in teaching music.
c. The teacher who advised the principals that Anne respected has no interest in teaching music.
d. The teacher who advised the principals that Anne respected have no interest in teaching music.

52. a. The secretary who clearly ignored the clients in the lobby threatens to quit her job twice a week.
b. The secretary who clearly ignored the clients in the lobby threaten to quit her job twice a week.
c. The secretary who ignored the clients that Thomas sent threatens to quit her job twice a week.
d. The secretary who ignored the clients that Thomas sent threaten to quit her job twice a week.

53. a. The minister who kindly helped the farmers at the town fair is eager to meet new people.
b. The minister who kindly helped the farmers at the town fair are eager to meet new people.
c. The minister who helped the farmers that Mary visited is always eager to meet new people.
d. The minister who helped the farmers that Mary visited are always eager to meet new people.

54. a. The prisoner who seriously injured the policemen in the fight has a history of being violent.
b. The prisoner who seriously injured the policemen in the fight have a history of being violent.
c. The prisoner who injured the policemen that Frank called has a history of being violent.
d. The prisoner who injured the policemen that Frank called have a history of being violent.

55. a. The speaker who warmly greeted the guests at the talk insists on meeting everyone in the audience.
b. The speaker who warmly greeted the guests at the talk insist on meeting everyone in the audience.
c. The speaker who greeted the guests that Carol invited insists on meeting everyone in the audience.
d. The speaker who greeted the guests that Carol invited insist on meeting everyone in the audience.

56. a. The witness who easily trusted the jurors in the room acknowledges that she didn't see the thief.
b. The witness who easily trusted the jurors in the room acknowledge that she didn't see the thief.
c. The witness who trusted the jurors that James selected acknowledges that she didn't see the thief.
d. The witness who trusted the jurors that James selected acknowledge that she didn't see the thief.

57. a. The researcher who rarely sent the doctors to the clinic creates reports for the governor.
b. The researcher who rarely sent the doctors to the clinic create reports for the governor.
c. The researcher who sent the doctors that Megan trusted creates reports for the governor.
d. The researcher who sent the doctors that Megan trusted create reports for the governor.

58. a. The student who truly liked the professors in the program studies for many hours every night.
b. The student who truly liked the professors in the program study for many hours every night.
c. The student who liked the professors that Tony fired studies for many hours every night.
d. The student who liked the professors that Tony fired study for many hours every night.

59. a. The therapist who never consulted the psychologists on staff refuses help from other professionals.

b. The therapist who never consulted the psychologists on staff refuse help from other professionals.
c. The therapist who consulted the psychologists that Tara saw refuses help from other professionals.
d. The therapist who consulted the psychologists that Tara saw refuse help from other professionals.

60. a. The nominee who clearly admired the investors at the fundraiser loves people with a lot of money.
b. The nominee who clearly admired the investors at the fundraiser love people with a lot of money.
c. The nominee who admired the investors that Mark recruited loves people with a lot of money.
d. The nominee who admired the investors that Mark recruited love people with a lot of money.

61. a. The visitor who slowly approached the generals from Washington distrusts anyone in a uniform.
b. The visitor who slowly approached the generals from Washington distrust anyone in a uniform.
c. The visitor who approached the generals that Nick guarded distrusts anyone in a uniform.
d. The visitor who approached the generals that Nick guarded distrust anyone in a uniform.

62. a. The scientist who just hired the technicians to the project develops the plans for deadly weapons.
b. The scientist who just hired the technicians to the project develop the plans for deadly weapons.
c. The scientist who hired the technicians that Rick called develops the plans for deadly weapons.
d. The scientist who hired the technicians that Rick called develop the plans for deadly weapons.

63. a. The editor who wildly hated the reporters in the office submits fake reports to the agency.
b. The editor who wildly hated the reporters in the office submit fake reports to the agency.
c. The editor who hated the reporters that Claire suspected submits fake reports to the agency.
d. The editor who hated the reporters that Claire suspected submit fake reports to the agency.

64. a. The widow who bravely rescued the teenagers from the fire attracts attention everywhere in town.
b. The widow who bravely rescued the teenagers from the fire attract attention everywhere in town.
c. The widow who rescued the teenagers that Adam brought attracts attention everywhere in town.
d. The widow who rescued the teenagers that Adam brought attract attention everywhere in town.
